# Fenestral diaphragms and PLVAP associations in liver sinusoidal endothelial cells are developmentally regulated

**DOI:** 10.1038/s41598-019-52068-x

**Published:** 2019-10-30

**Authors:** Kaisa Auvinen, Emmi Lokka, Elias Mokkala, Norma Jäppinen, Sofia Tyystjärvi, Heikki Saine, Markus Peurla, Shishir Shetty, Kati Elima, Pia Rantakari, Marko Salmi

**Affiliations:** 1 MediCity Research Laboratory, University of Turku, Turku, Finland; 2 Institute of Biomedicine, University of Turku, Turku, Finland; 3 Centre for Liver Research, Institute of Immunology and Immunotherapy, University of Birmingham, Birmingham, United Kingdom

**Keywords:** Angiogenesis, Differentiation, Cardiovascular biology, Hepatology

## Abstract

Endothelial cells contain several nanoscale domains such as caveolae, fenestrations and transendothelial channels, which regulate signaling and transendothelial permeability. These structures can be covered by filter-like diaphragms. A transmembrane PLVAP (plasmalemma vesicle associated protein) protein has been shown to be necessary for the formation of diaphragms. The expression, subcellular localization and fenestra-forming role of PLVAP in liver sinusoidal endothelial cells (LSEC) have remained controversial. Here we show that fenestrations in LSEC contain PLVAP-diaphragms during the fetal angiogenesis, but they lose the diaphragms at birth. Although it is thought that PLVAP only localizes to diaphragms, we found luminal localization of PLVAP in adult LSEC using several imaging techniques. *Plvap*-deficient mice revealed that the absence of PLVAP and diaphragms did not affect the morphology, the number of fenestrations or the overall vascular architecture in the liver sinusoids. Nevertheless, PLVAP in fetal LSEC (fenestrations with diaphragms) associated with LYVE-1 (lymphatic vessel endothelial hyaluronan receptor 1), neuropilin-1 and VEGFR2 (vascular endothelial growth factor receptor 2), whereas in the adult LSEC (fenestrations without diaphragms) these complexes disappeared. Collectively, our data show that PLVAP can be expressed on endothelial cells without diaphragms, contradict the prevailing concept that biogenesis of fenestrae would be PLVAP-dependent, and reveal previously unknown PLVAP-dependent molecular complexes in LSEC during angiogenesis.

## Introduction

Vascular endothelium is a critical gate-keeper between the circulation and different tissue environments. Under normal conditions it regulates the development, homeostasis and metabolism of tissues, and under pathological conditions it is heavily involved in inflammation and tumorigenesis^1–3^. Heterogeneity of vascular endothelium has been recognized for decades^4^. The endothelial cells (EC) are divided to continuous, fenestrated and sinusoidal based on their subcellular morphology^4,5^. Continuous EC, the most common endothelial type, are barrier forming cells, which allow diffusion of water and small solutes to the extravascular compartment without loss of plasma proteins or blood cells. The barrier function of EC is particularly pronounced in some highly specialized vascular beds, like at the blood-brain barrier. Fenestrated EC, on the other hand, have intracellular openings, which penetrate the whole thickness of the cell. These fenestrae (open pores, “windows”) are usually covered by a thin proteinaceous diaphragm. This capillary type is characteristic to specific organs, like kidney, several endocrine organs and choroid plexus, which display increased permeability. Finally, the sinusoidal discontinuous endothelium is found in liver, spleen and bone marrow. Larger fenestrations, incomplete basement membrane, and possibly interendothelial gaps, specify this EC type.

The only known protein component in the diaphragms covering the endothelial fenestrations is PLVAP^6–10^. This protein, also known as PV-1, PAL-E and MECA-32 antigen, is a type 2 transmembrane glycoprotein, which forms rod-like homodimeric fibrils that are thought to radiate from the rim of the fenestration towards a central knob^7,11^. When analyzed from kidney and pancreas EC, the diaphragms displayed a cart-wheel like structures, which typically show a 50–70 nm diameter, although the range of diaphragms varied from <50 nm to >93 nm, and the size distribution was partially affected by tissue fixation methods used^12^. PLVAP-diaphragms also guard transendothelial channels both at the luminal and abluminal aspects of the EC^6^. In addition PLVAP-diaphragms are present on a subset of caveolae, which are flask-shaped invaginations of the plasma membrane serving multiple endocytic and signaling functions^6,13^. Notably, according to the current dogma, PLVAP is thought to be present only in diaphragms^7–9^.

The physiological functions of PLVAP have been analyzed in three independently generated *Plvap*^−/−^ mouse lines^8,9,14^. The absence of PLVAP results in high embryonic lethality with only low numbers of surviving pups in mixed backgrounds. The newborns are growth retarded and survive only about 5–6 wk. In all three mouse lines, deletion of PLVAP results in loss of all identifiable diaphragms. While PLVAP is necessary for diaphragm formation, it is not sufficient for it. This was shown in elegant experiments by Stan *et al*. showing that forced pan-endothelial PLVAP expression in *Plvap*^−/−^ mice did not result in diaphragm formation in those vascular beds in which PLVAP is not normally expressed^8^. *Plvap*^−/−^ mice manifest with increased vascular permeability resulting in leakage of intravascular proteins smaller than 70 kDa out from the vasculature^8^. *Plvap*^−/−^ mice are thought to succumb due to protein losing enteropathy^8^, and a similar fatal outcome has been reported in  four human patients lacking intact PLVAP^15–17^. In addition, in mice PLVAP regulates the permeability of subcapsular sinus lymphatic endothelial cells to leukocytes and antigens in peripheral lymph nodes^14^, and the generation of fetal-derived macrophages in the fetal liver^18^.

Contradicting reports have been published regarding PLVAP expression and functions in the liver^8,11,19–24^. The liver vasculature has a complex architecture^5,25^. Oxygenated blood from the hepatic artery and intestine-draining blood from the portal vein enter the liver within the portal tracts, mix in the liver sinusoids percolating between the hepatocyte cords, and leave the organ via central venules and finally via the hepatic vein. Liver sinusoids show zonation along the portal to central vein axis, which also reflected in the phenotype of liver sinusoidal endothelial cells (LSEC)^26–29^. Characteristic to LSEC are numerous fenestrations, which are largely concentrated to sieve plates^30–33^. The fenestrations (open pores, “windows”) have a mean diameter of ~150–175 μm (range ~75–300 μm) in rat LSEC when measured by transmission electron microscopy, but the reported dimensions vary depending on the species, age, sinusoidal zonation and method of analyses^29–31,34,35^. The density of fenestrations also differs in the different sinusoidal zones^29,35^. The fenestrations allow free contacts between small blood components (e.g. chylomicron remnants), and hepatocytes and stellate cells through the narrow space of Disse^31,32^. Early electron microscopy studies have shown the presence of diaphragms in fetal rat sinusoids^36,37^ and the lack of diaphragms in the adult sinusoids^29,30,34,35^. LSEC also express caveolin-1, an essential component of caveola^38^. A very high endocytic capacity is another typical feature of LSEC.^30–33^. In fact, LSEC show a clear division of work with macrophages in actively scavenging blood-borne macromolecular waste molecules and immune complexes^39,40^.

Consistent with the lack of diaphragms in the adult liver LSEC.^31–33,35^, many studies have reported that PLVAP is absent from normal LSEC.^19–22^. However, others have found variable PLVAP protein or mRNA expression in the adult liver^7,23,24^ and it has been claimed that PLVAP is actually needed for the fenestral biogenesis^24^. Therefore, our aim was to explore the synthesis of PLVAP, diaphragms and fenestrations in the different EC compartments (arterial, portal vein, sinusoidal and central vein) of liver, analyze PLVAP associations in LSEC and re-examine the functional role of PLVAP in fenestral biogenesis *in vivo*. Using multimodal imaging and PLVAP-deficient mice we show that while not necessary for fenestral biogenesis, PLVAP forms diaphragms and is associated with angiogenic signaling complexes during fetal liver angiogenesis, whereas in adult LSEC it displays a conceptually novel extra-diaphragmatic localization.

## Results

### Prenatal LSEC have PLVAP-diaphragms in fenestrae

**Figure 1:**
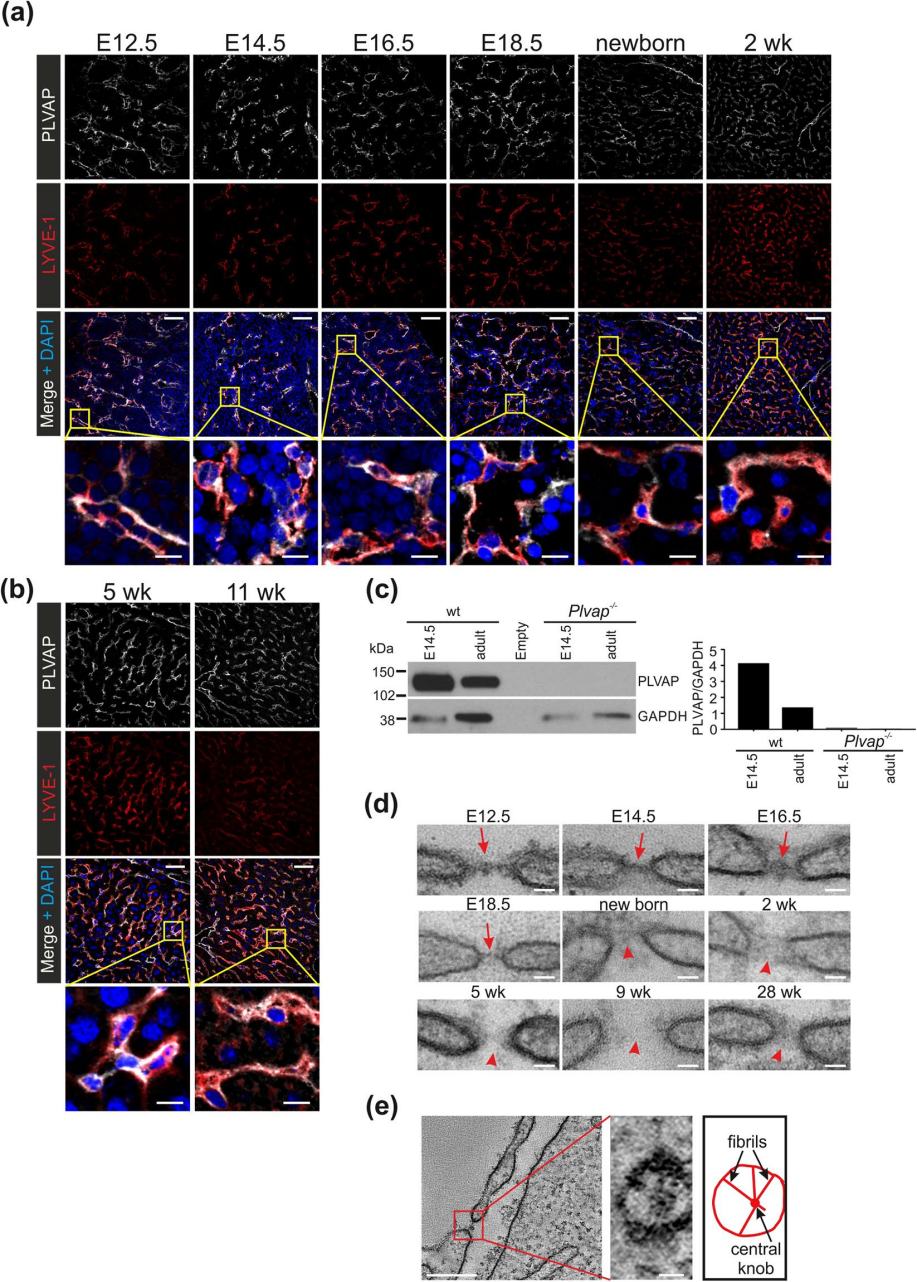
Fenestral diaphragms and PLVAP expression in LSEC during liver ontogeny. (**a,b**) Confocal microscopy analyses of PLVAP and LYVE-1 expression in LSEC during the fetal (E12.5-E18.5) and postnatal (newborn-11 wk) development. Bars, 50 µm (main figures) and 10 µm (insets). (**c**) Immunoblot analyses of fetal and adult liver lysates (5 µg protein/lane) from wild-type (wt) and *Plvap*^−/−^ mice for PLVAP and GAPDH (a house keeping molecule) under non-reducing conditions. The same filter was sequentially probed for PLVAP and GAPDH. Full blots are shown in Suppl. Figure 10. (**d**) Transmission electron micrographs from LSEC fenestra in fetal and postnatal liver at the indicated ages. Red arrows, diaphragms; red arrowheads, open fenestra. Bars, 40 nm. (**e**) Electron tomography of a diaphragm in E14.5 fetal liver of wild-type mice. Perpendicular view of the boxed area is shown at a higher magnification in the inset. Bars, 200 nm (inset 20 nm). Shown are representative images (numbers of mice/genotype: n = 3 (**a**,**b**), n = 1 (E16.5, 9 wk, 28 wk in **d**,**e**), n = 2 (E14.5 wild-type in **c**, and E12.5 in **d**), n = 3–4 (E14.5, E18.5, new-born, 2 wk and 5 wk in **d**), n = 4 (adult wild-type, and E14.5 and adult *Plvap*^−/−^ in **c**).

Since there are discrepant reports regarding the expression of PLVAP protein in liver^8,11,19–24^, we used timed matings to get fetal and postnatal liver specimens from wild-type mice (here all wild-type mice refer to littermate controls of *Plvap*^−/−^ mice, unless otherwise stated) for re-evaluating the expression of PLVAP in LSEC during liver angiogenesis. Using an anti-PLVAP mAb MECA-32 and confocal microscopy analyses we found that PLVAP was expressed in virtually all LSEC (defined by LYVE-1 expression^31,41,42^) before birth (Fig. 1a). Clear PLVAP-positivity in LYVE-1^+^ LSEC was also found in new-born mice, as well as at later postnatal time points (Fig. 1a,b). No apparent PLVAP expression was found in non-vascular cell types in liver at any developmental stage studied. In addition to these hybrid littermate wild-types, PLVAP protein in LSEC was found also in all other mouse strains studied (Balb/c, C57BL6J, and C57BL6N; Suppl. Figure 1a,b).

Consistent with PLVAP mRNA analyses in adult mouse liver^8,23,24^, our immunoblotting analyses confirmed that PLVAP protein was present in adult liver lysates and revealed that it migrated as a ~120 kDa band consistent with a homodimeric nature of the molecule (Fig. 1c). The quantifications of PLVAP/GAPDH ratios implied that relatively more PLVAP may be present in liver at E14.5 embryos than in adults (Fig. 1c), with the caveat that the identification of a stable loading control protein in a developing complex organ is inherently challenging. The specificity of the MECA-32 antibody was confirmed by the lack of reactivity with liver lysates of *Plvap*^−/−^ mice. Thus, PLVAP protein is expressed in the liver throughout the development, and it localizes to vessels.

In line with earlier reports in rats and mice^24,36,37^, we found typical cross-sectional profiles of protein diaphragms overlaying LSEC fenestrations throughout the embryonic development (E12.5-E18.5) (Fig. 1d). Electron tomography showed that the diaphragms in the fetal LSEC displayed the characteristic radial fibrils spanning from the fenestral rim towards a central density (Fig. 1e). Notably, after birth (postnatal day 0.5, and 5 wk, 9 wk and 28 wk old mice), no identifiable diaphragms were observed any more in the sinusoidal fenestrations (Fig. 1d), as previously reported by others^29,30,34,35^.

Our electron microscopy data is in line with the prevailing concept that the adult sinusoidal vasculature in liver contains open fenestrations, but that LSEC fenestrations are guarded by diaphragms until the birth. Collectively, our data show that PLVAP protein in the LSEC is induced early on, expressed throughout the liver angiogenesis and maintained in the adulthood.

### Non-diaphragmal PLVAP in adult LSEC

**Figure 2:**
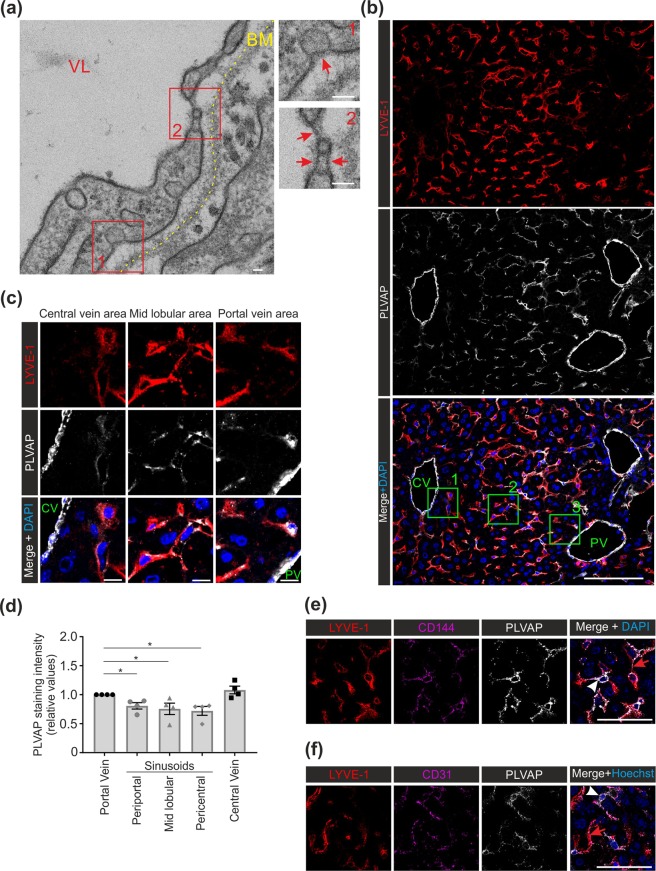
PLVAP expression in the diaphragmed and non-diaphragmed domains of endothelial cells in adult liver. (**a**) Transmission electron micrographs from a vein in liver of a 9 wk old wild-type mouse. VL, vascular lumen, BM, basement membrane (yellow dotted line). The two boxed areas are shown at a higher magnification in the insets. Red arrows, diaphragms in caveolae, fenestrae, and transendothelial channels. Bar, 40 nm. (**b**) Confocal microscopy analyses of PLVAP and LYVE-1 expression in 5 wk old wild-type mice. Blue is DAPI. CV, central venule; PV, portal venule. Bar, 100 µm. (**c**) Higher magnifications of the areas defined by the green boxes in (**b**) Bar, 10 µm. (**d**) Quantification of relative PLVAP staining intensities (mean ± s.e.m, n = 4 mice) from the portal vein, periportal, midlobular and pericentral sinusoids and from the central vein. Intensity value 1.0 was assigned to the portal vein in each animal. (**e**,**f**) Confocal microscopy analyses of PLVAP, LYVE-1 and CD144 (**e**) and PLVAP, LYVE-1 and CD31 (**f**) expression in 5 wk old wild-type mice. White arrowheads point to LYVE-1^−^PLVAP^+^CD144^+^ (**e**) and LYVE-1^−^PLVAP^+^CD31^+^ (**f**) sinusoids. Red arrowheads point to LYVE-1^+^PLVAP^+^CD144^+^ (**e**) and LYVE-1^+^PLVAP^+^CD31^+^ sinusoids (**f**). Bars, 50 µm. Shown are representative images (numbers of mice/genotype: n = 3 (**a,e,f**); n = 4 (**b,c**)). *p < 0.05.

To address the unexpected finding of PLVAP expression in the absence of fenestral diaphragms in adult LSEC, we further analyzed the relation of diaphragms and PLVAP in the liver vasculature. Electron microscopy showed that diaphragms were indeed still present in larger veins of adult liver in wild-type mice. These vein EC, underlined by a well-developed basement membrane, displayed typical caveolae, fenestrations and transendothelial channels, all of which had clear diaphragms (Fig. 2a, Suppl. Figure 1c). In 5 wk old *Plvap*^−/−^ mice, the vein EC also contained uncoated endocytic carriers (possibly caveolae) and fenestrations or transendothelial channels, but they lacked all diaphragms (Suppl. Figure 1d,e).

In confocal microscopy analyses of the vascular segments in adult liver, the portal and central vein EC were PLVAP bright, whereas no signal compatible with the PLVAP expression in the hepatic artery was detectable (Fig. 2b). The identity of these non-sinusoidal vessels was confirmed in additional experiments by histology (hematoxylin-eosin stainings) and by including CD31 as a pan-endothelial cell marker. These stainings and their quantification confirmed PLVAP-negativity of the hepatic artery (Suppl Figure 2a–c). We found that the LSEC in the periportal, pericentral segments, and mid-lobular sinusoid segments all expressed PLVAP (Fig. 2b,c). When quantified, the PLVAP expression level was lower at all sinusoidal locations when compared to the portal vein (Fig. 2d, Suppl. Figure 2d). Notably, some LSEC, which were LYVE-1^neg/low^, were clearly PLVAP^+^ (Fig. 2e,f). The LYVE-1^neg/low^PLVAP^+^ vessels also expressed CD144 (VE-cadherin) and CD31 (Fig. 2e,f, and Suppl. Figure 2e,f), confirming their vascular identity. Our observations of strong PLVAP expression in the venular EC are in line with our electron micrographs revealing the presence of diaphragmed caveolae, fenestrations and transendothelial channels in these vessels. However, the stainings also showed definitive PLVAP expression in the majority of LSEC in adults.

**Figure 3:**
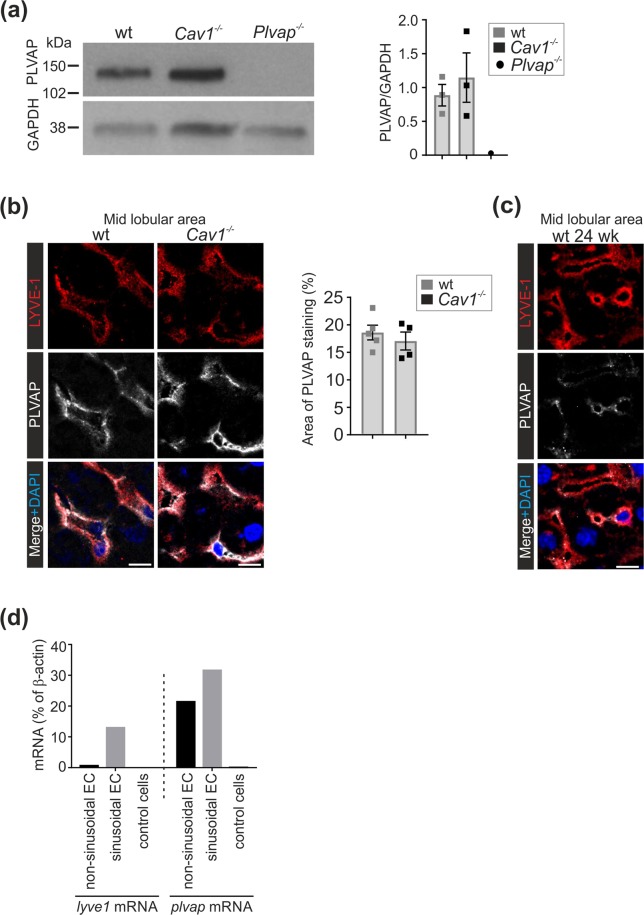
Expression of PLVAP in *Cav1*-deficient liver. (**a**) Immunoblot analyses of adult liver lysates (5 µg protein/lane) from 5 wk old wild-type, *Cav1*^−/−^ and *Plvap*^−/−^ mice for PLVAP and GAPDH (a house keeping molecule) under non-reducing conditions. Quantitative data are mean ± s.e.m. (n = 3 mice/genotype). The same filter was sequentially probed for PLVAP and GAPDH. Full blots are shown in Suppl. Figure  10. (**b**) Confocal microscopy analyses of PLVAP and LYVE-1 expression in the midlobular area of 5 wk old wild-type and *Cav1*^−/−^ mice and quantification of the PLVAP^+^ area (mean ± s.e.m, each dot represents one mouse). Bars, 10 µm. (**c**) Analyses of PLVAP expression in 24 wk old wild-type mice. Bars, 10 µm. (**d**) Quantitative PCR analyses of *Lyve-1* and *Plvap* mRNA expression in sorted sinusoidal ECs (CD45^−^Podoplanin^−^LYVE-1^+^CD144^+^ cells) and non-sinusoidal (CD45^−^Podoplanin^−^LYVE-1^−^CD144^+^) EC and leukocytes (control cells) (n = 5 mice for both cell types). β-actin was used as a control gene. Shown are representative images (numbers of mice/genotype: n = 3(**a**), n = 4–6 (**b**), n = 1 (**c**).

LSEC are reported to contain low levels of caveolae^38^, which can be overlaid by PLVAP-diaphragms in other organs^6^. To study the potential role of caveolae in conferring PLVAP-positivity to the sinusoidal segments of adult liver vasculature we used Caveolin-1(*Cav-1*)-deficient mice, which lack all caveolae^43^. Using immunoblotting, a clear PLVAP signal was evident from the liver lysates of *Cav1*^−/−^ mice (Fig. 3a). Confocal microscopy further showed that in the absence of caveolae PLVAP was still expressed in the liver sinusoids (Fig. 3b, Suppl. Figure 3a–c). Thus, caveolae and their diaphragms in LSEC have minimal, if any, contribution to the PLVAP-positivity of adult LSEC.

To interrogate the possibility that PLVAP protein in adult LSEC would present a carry-over from the fetal period, we analyzed its persistence in old mice and evaluated *Plvap* mRNA synthesis in different EC populations in adult liver. We found clear PLVAP positivity in LSEC of 24 wk old wild-type mice (Fig. 3c and Suppl. Figure 3d,e), which argues against the carry-over effect. We then sorted sinusoidal and non-sinusoidal blood vascular EC^44^ from livers of 5 wk old wild-type mice. CD45 and podoplanin were used to exclude leukocytes and lymphatic EC, respectively. CD144^+^ EC were then divided into LYVE1^+^ sinusoidal and LYVE1^−^ non-sinusoidal (venular and arterial EC). As expected, *Lyve-1* mRNA was present only in the sinusoidal EC compartment, and not in the large vessel EC or in an irrelevant control cell type (leukocytes) (Fig. 3d) verifying the purity of the sorted EC populations. Analyzing these cell populations for *Plvap* mRNA, we found robust and specific synthesis both in the large vessel and sinusoidal EC in the adult liver (Fig. 3d). Collectively these data show that adult LSEC synthesize *Plvap* mRNA and express PLVAP protein in non-caveolar structures.

### Cell surface expression of PLVAP in adult LSEC

**Figure 4:**
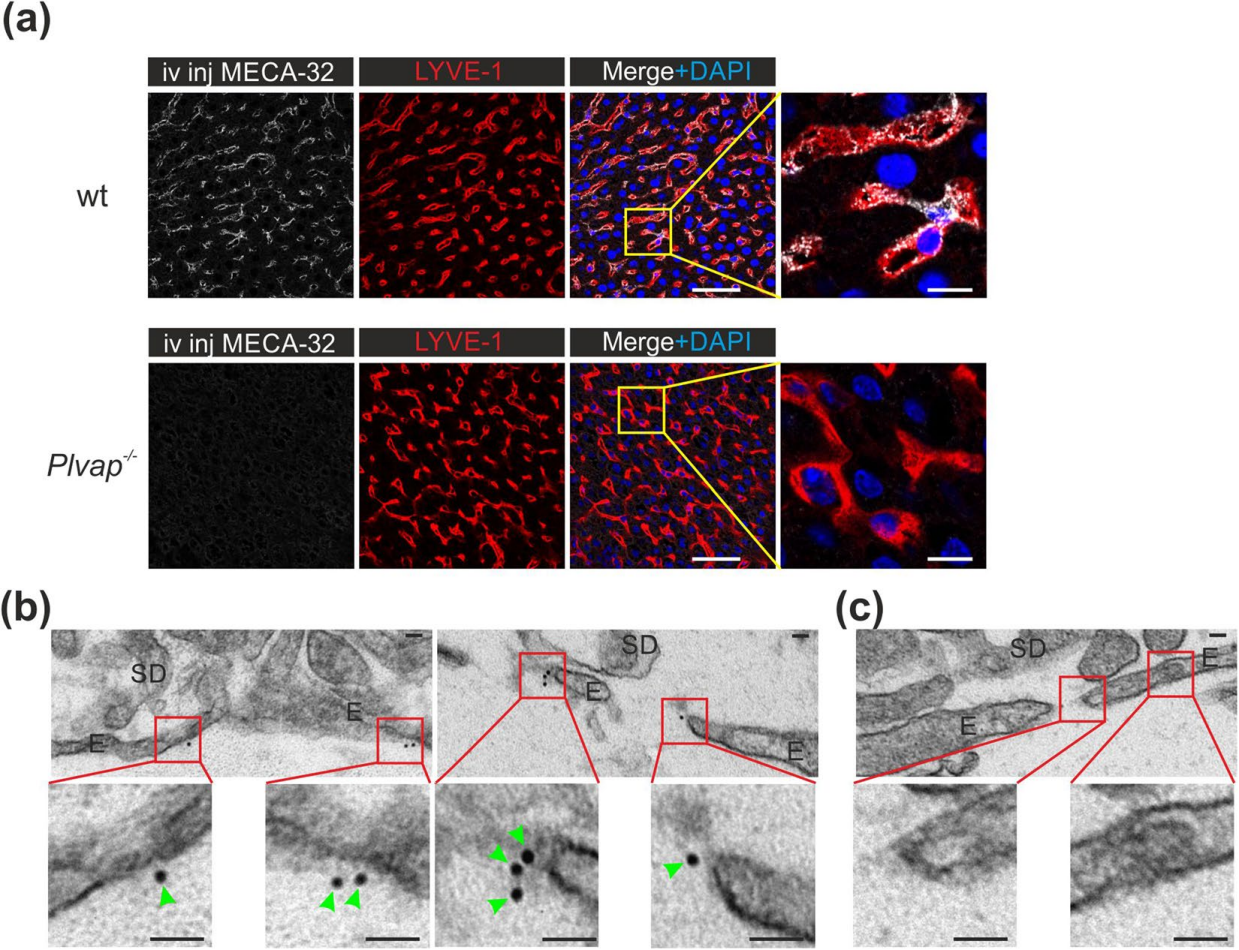
Cell-surface expression of PLVAP in adult LSEC in the absence of diaphragms. (**a**) Confocal microscopy analyses of LSEC in 5 wk old wild-type and *Plvap*^−/−^ mice 10 min after an intravenous bolus of the anti-PLVAP antibody (MECA-32). LYVE-1 was stained *ex vivo*. Bars, 50 µm (main figures), 10 µm (insets). (**b**,**c**) Immuno-electron micrographs of MECA-32 (**b**) and rat IgG2a isotype control antibody (**c**) stained liver sections after detection with an anti-rat immunoglobulin conjugated to 10 nm gold particles. Higher magnifications of the boxed areas are shown in the insets. E, sinusoidal endothelial cell; SD, space of Disse. Green arrowheads point to gold particles. Bars, 40 nm. Shown are representative images (numbers of mice/genotype: n = 3 (**a**, wild-type), n = 1 (**a**, *Plvap*^−/−^), n = 3 (**b**,**c**)).

To analyze if the PLVAP protein in the absence of diaphragms would be mistargeted to intracellular compartments in the adult LSEC (similar to CD31^45^), we performed *in vivo* labelings of the liver vasculature. When the anti-PLVAP antibody (MECA-32) was administered intravenously to wild-type mice, a specific signal was recovered 10 min later from the LYVE1^+^ LSEC (Fig. 4a). Similar MECA-32 injections into the *Plvap*-deficient mice gave no signal in LSEC or in any other cell type verifying the specificity of the approach (Fig. 4a).

We then used immunoelectron microscopy to visualize PLVAP localization in adult LSEC. The results were compatible with the possibility that PLVAP is expressed on the luminal plasma membrane of sinusoidal vessels (Fig. 4b and Suppl. Figure 4a). PLVAP signal was detected both from the membrane areas juxtapositioned to the fenestral pores and from the plasma membrane area that was apparently separated from the fenestrations. No PLVAP-signal was detected in non-EC types, and an isotype matched control antibody gave no signal in fenestral or non-fenestral area of EC or in any other cell type (Fig. 4c and Suppl. Figure 4a,b). Moreover, after *in vivo* injection of gold-labelled MECA-32 antibody, the gold nanoparticles were found on the sinusoidal EC membrane (Suppl. Figure 4c). Within the technical limitations of the assays (e.g. possible internalization of the circulating MECA-32 antibody, resolution limits of the two-stage gold labeling technique, physicochemical properties of the directly gold-labeled MECA-32 antibody) these analyses suggest that despite the absence of fenestral diaphragms in adult liver sinusoids, PLVAP protein is present on the surface of LSEC.

### PLVAP is not needed for the formation of fenestrations or sinusoids in the liver

**Figure 5:**
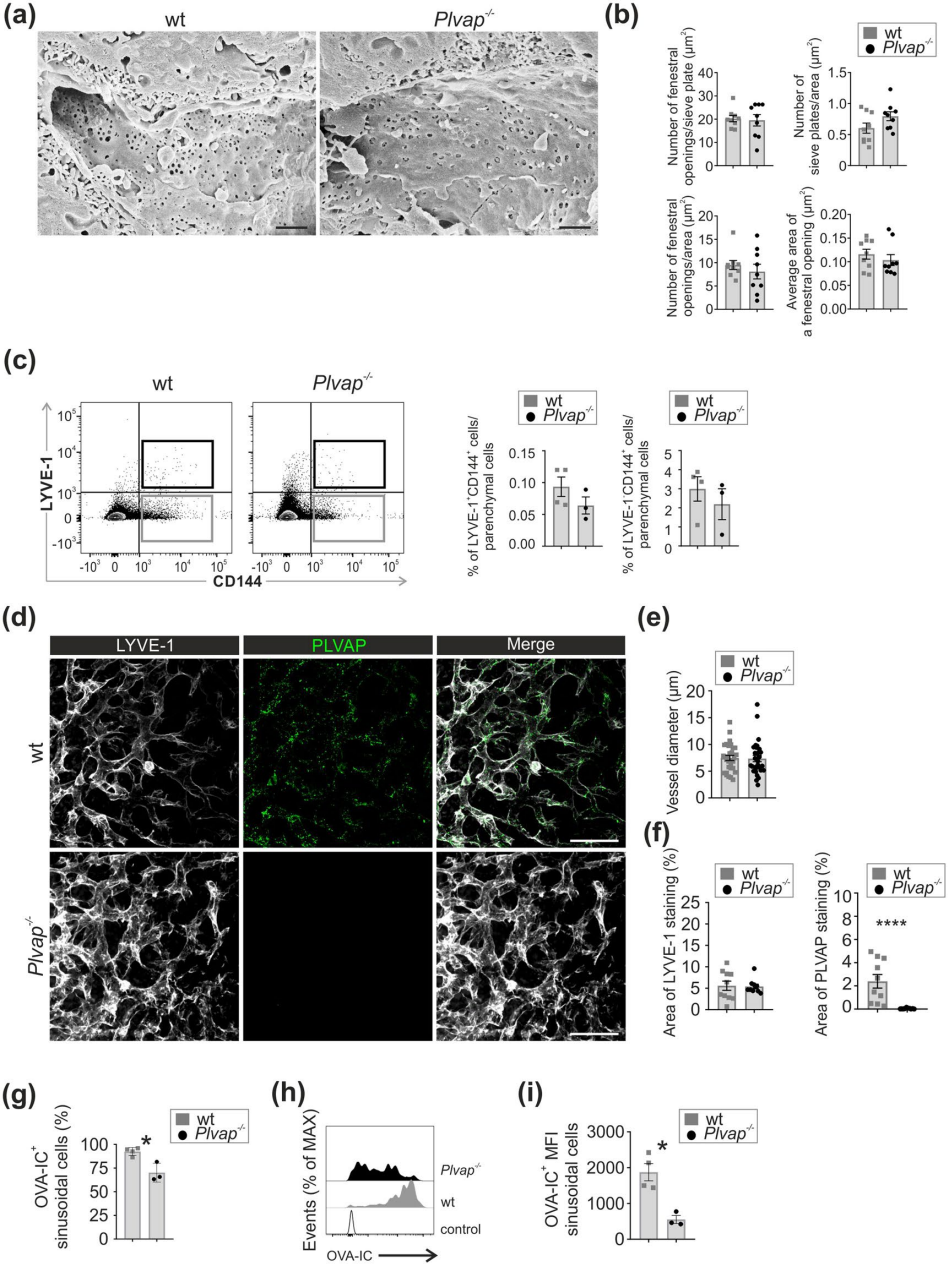
Absence of PLVAP does not affect fenestral formation or liver angiogenesis but alters immunocomplex binding to LSEC. (**a**) Scanning electron micrographs of sinusoidal vessels in 5 wk old wild-type and *Plvap*^−/−^ mice. Bars, 1 µm. (**b**) Quantification (mean ± s.e.m., n = 9, 3 field/mouse, 3 mice/genotype) of the number of fenestral openings per sieve plate, the number of sieve plates per endothelial surface area, the number of fenestrations per endothelial cell area and the average surface area of a fenestral opening. (**c**) Flow cytometric analysis of liver EC (LYVE-1^+^CD144^+^ LSEC) and their quantification per CD45^−^ parenchymal cells (left) and LYVE-1^−^CD144^+^ non-sinusoidal ECs per CD45^−^ parenchymal cells (right)) in 5 wk old wild-type and *Plvap*^−/−^ mice. Quantitative data are mean ± s.e.m. (n = 3–4 mice/genotype). (**d**) Maximal z-stack projections (48 µm) of whole-mount stainings of E14.5 liver from wild-type and *Plvap*^−/−^ mice for LYVE-1 and PLVAP. Bars, 50 µm. (**e**), Quantification of sinusoidal vessel diameters in wild-type and *Plvap*^−/−^ mice (mean ± s.e.m, 30 vessels/genotype, 2 mice/genotype). (**f**) Quantification of LYVE-1 and PLVAP signal areas from single optical sections (mean ± s.e.m, 10 vessels/genotype, 2 mice/genotype). (**g**–**i**) Flow cytometric analyses of LSEC (Live^+^CD45^−^LYVE-1^+^CD144^+^ cells) 2 h after an intravenous injection of Atto-488 conjugated OVA-IC in 5 wk old wild-type and *Plvap*^−/−^ mice. (**g**) The percentage of LSEC (from all LSEC) that have taken up OVA-IC (mean ± s.e.m, 3–4 mice/genotype). (**h**) Representative histograms of the distribution of Atto-488-labeled OVA-IC in LSEC. Control, a mouse to which vehicle only (PBS) is administered. **i** Quantification of the mean fluorescence intensities (MFI) from (**g**). Representative images are from (n = 3 mice/genotype (**a**), and n = 2 mice/genotype (**d**) and 3–4 mice/genotype (**h**). *p < 0.05, ***p < 0.001.

Since fetal LSEC fenestrations were covered by diaphragms and since the generation of fenestrae in liver has been reported to be PLVAP-dependent^24^, we visualized fenestrations by scanning electron microscopy. In adult wild-type mice LSEC fenestrations grouped into the typical sieve plates (Fig. 5a). In *Plvap*^−/−^ mice fenestrations (or transendothelial channels, which cannot be separated from fenestrations in the absence of diaphragms) were also readily detectable (Fig. 5a). Based on the quantifications of the areas covered by sieve plates, the numbers of fenestrations within the sieve plates, the number of fenestrae/ µm^2^ endothelial surface area and the average size of fenestral pores, the fenestrations were morphologically similar in *Plvap*^−/−^ and wild-type mice (Fig. 5b). The sinusoids in *Plvap*^−/−^ mice looked morphologically grossly normal (single EC layer, typical intracellular organelles, intact inter-endothelial junctions, no basement membrane) also in all TEM analyses. The only exception was a small reduction in the width of space of Disse in *Plvap*^−/−^ mice (Suppl. Figure 5a).

We then studied the general sinusoidal architecture in the adult liver in the absence of PLVAP. Immunofluorescence analyses of liver sections revealed apparently similar sinusoidal architecture and sinusoidal LYVE-1 (a marker for sinusoidal and lymphatic vessels) and CD31 (a pan-EC marker) expression in the adult wild-type and *Plvap*^−/−^ mice (Suppl. Figure 5b–e). Quantifications verified the similar CD31-positive EC area in both genotypes (Suppl. Figure 5b). Additional quantitative flow cytometric analyses showed that the percentages of both LYVE-1^+^CD144^+^ sinusoidal ECs and LYVE-1^−^CD144^+^ large vessel EC were comparable in the adult livers of wild-type and *Plvap*^−/−^ mice (Fig. 5c, Suppl. Figure 5f). Moreover, 3D-analyses of E14.5 livers, amenable to whole-mount imaging after optical clearing, showed similar sinusoid diameters and LYVE-1^+^ vascular area in actively angiogenic livers of wild-type and *Plvap*^−/−^ mice (Fig. 5d–f and Suppl. Video 1). In scanning electron microscopy analyses of non-perfused E17.5 embryos putative open fenestrations were found in vessels of both genotypes (Suppl. Figure 5g). Collectively these data show that PLVAP protein or diaphragms at any developmental stage are not necessary for the formation of fenestrations or for the normal sinusoidal angiogenesis in the liver.

### Altered scavenging of immunocomplexes in the liver of *Plvap*^−/−^ mice

We studied if the genetic absence of PLVAP could lead to alterations in endocytosis, which is one of the characteristic functions of LSEC. We administered fluorescent beads (20 nm and 0.5 µm), acetylated LDL or ovalbumin (OVA)-anti-OVA antibody immunocomplexes (OVA-IC) intravenously to wild-type and *Plvap*^−/−^ mice. Using microscopy we found that all four ligands were bound/taken up by both LYVE1^+^ cells (presumably LSEC) and F4/80^+^ cells (presumably Kupffer cells and other macrophages) in the liver in both wild-type and *Plvap*^−/−^ mice (Suppl. Figure 6). Since cell type identification benefits from the use of multiple markers, and since intimate contacts between LSEC and intravascular Kupffer cells make it difficult to quantify signals separately from these two cell types, we chose to analyze the up-take of OVA-IC in more detail by processing livers from the *in vivo* OVA-IC-treated mice for flow cytometric analyses. In line with our previous observations^18^ we found a strong reduction in the numbers of CD45^+^CD11b^low^F4/80^high^ Kupffer cells, and possibly a slight increase in the numbers of other liver-resident macrophages (CD45^+^CD11b^high^F4/80^intermediate^) in the absence of PLVAP (Suppl Fig. 7a,b). In wild-type mice about 40% of both macrophage types bound OVA-IC. The remaining Kupffer cells, but not the dominant non-Kupffer macrophages, showed significantly reduced OVA-IC scavenging in *Plvap*^−/−^ mice (Suppl. Figure 7c–e). When focusing on endothelial scavenging function, the FACS analyses showed that practically 100% of LSEC in the wild-type mice had efficiently bound or taken up OVA-IC (Fig. 5g). In *Plvap*^−/−^ mice the amount of OVA-IC ingested per LSEC was significantly reduced (Fig. 5h,i). It should be noted that administration of the same amount of OVA-IC to wild-type and *Plvap*^−/−^ mice results in some uncertainty regarding the availability of the OVA-IC to LSEC due to different macrophage numbers, altered blood composition, and organ size in the absence of PLVAP. Similar to the other reported *Plvap*^−/−^ lines^8,9^, our *Plvap*^−/−^ mice show modest reduction of blood erythrocytes in the presence of unaltered numbers of other blood cell types, hyperlipemia and reduced concentration of blood proteins smaller than 70 kDa (Suppl. Figure 8a–c). Our *Plvap*^−/−^ mice or their livers were not severely growth retarded, and their liver/body weight ratio was actually somewhat increased when compared to littermate controls (Suppl. Figure 8d–e). It is possible that genetic absence of PLVAP in the EC and/or the secondary effects of vascular barrier defects may alter the transcriptome of PLVAP-deficient EC (e.g. by affecting the expression of Fc- or scavenging receptors or endocytic machinery) and thereby lead to altered binding of immunocomplexes. Therefore, the presence of confounding factors needs to be taken into account when interpretating these scavenging data.

### PLVAP associates with different molecules in fetal and adult LSEC

**Figure 6:**
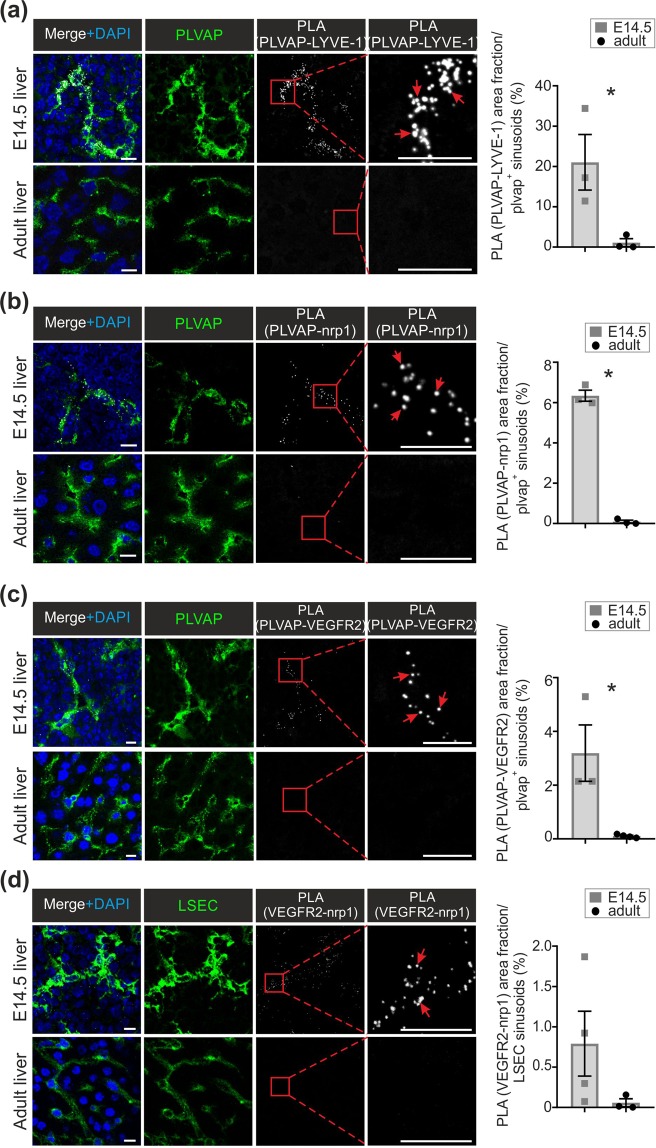
PLVAP associates with LYVE-1, neuropilin-1 and VEGFR2 in fetal but not adult LSEC. (**a–d**) Proximal ligation analyses (PLA) of the associations between PLVAP and LYVE-1 (**a**), PLVAP and neuropilin-1 (Nrp1) (**b**), PLVAP and VEGFR2 (**c**) and VEGFR2 and Nrp1 (**d**) in fetal and adult livers. The white signals show associations between the indicated molecules, and the green stainings show expression of PLVAP (**a–c**) and VEGFR2 and Nrp1 (**d**). The boxed areas are shown at a higher magnification in the insets. Red arrows point to representative PLA signals. Bars, 10 µm. The quantifications (the area fraction of the white signal per PLVAP (**a**–**c**) or Nrp1 + VEGFR2 (**d**) positive sinusoids) are shown (mean ± s.e.m., n = 2–5 fields/mouse, 3–4 mice/genotype). *p < 0.05.

In addition to homodimeric interactions necessary for diaphragm formation^6^, PLVAP may associate with other molecules in EC. To evaluate this, we used proximal ligation assays, which gives a positive signal if the two molecules associate (i.e. are less than 40 nm apart from each other^46^). We noticed that although both fetal and adult liver express PLVAP and LYVE-1, a PLA signal between the two molecules was only detectable in the fetal LSEC (Fig. 6a).

The possible molecular association between neuropilin-1 and PLVAP, which has been previously described in an adult non-hepatic diaphragmed vasculature^47^, was then studied. In wild-type mice we observed a clear expression of neuropilin-1 in E14.5 LSEC (Suppl. Figure 9a), and its association with PLVAP (Fig. 6b). In the adult wild-type LSEC, both PLVAP and neuropilin-1 (Suppl. Figure 9b) were still present, but the PLA signal was completely lost (Fig. 6b). The expression of neuropilin-1was not dependent on the association with PLVAP, since the distribution of neuropilin-1 was unaltered in fetal and adult LSEC in *Plvap*^−/−^ mice (Suppl. Figure 9a,b).

Since neuropilin-1 is an important co-receptor in VEGF-driven signaling^48^, we also studied the association of PLVAP and the main VEGF receptor, VEGFR2, in the liver sinusoids. We found a novel molecular association between PLVAP and VEGFR2 in the fetal LSEC (Fig. 6c). The association between the two molecules was not detectable in adult LSEC (Fig. 6c), although VEGFR2 was still present in the adult sinusoids (Suppl. Figure 9c). In fact, the association of neuropilin-1 and VEGFR2 seemed to display a similar developmental regulation, since it was seen mainly in the fetal liver (Fig. 6d). Collectively these data suggest that in the fetal LSEC PLVAP is engaged both in homodimeric interactions necessary for the formation of diaphragms^6^, and in molecular associations with the critical surface components of the VEGFR2-angiogenic signaling pathway. Concomitantly with the disappearance of PLVAP diaphragms at birth, PLVAP molecules on the LSEC membrane become molecularly separated from neuropilin-1 and VEGFR2 (and from Lyve-1), and the neuropilin-1-VEGFR2 complex dissociates.

## Discussion

Here we show that during the fetal development LSEC contain fenestrations covered with diaphragms, which are formed by PLVAP protein. Despite the disappearance of the diaphragms in LSEC, but not in vein ECs, at birth, LSEC continue to express PLVAP on the membrane in the adulthood. This is striking, since so far PLVAP has been thought to reside exclusively in the diaphragms^6,8^. Genetic absence of PLVAP, and hence of the fetal fenestral diaphragms, did not affect the generation of fenestrations or the vascular architecture in the liver. We also found novel associations between PLVAP and LYVE-1, neuropilin-1 and VEGFR2 in fetal LSEC.

The biogenesis of LSEC fenestrae remains poorly understood^31–33^. Typically 6–8% of LSEC surface area is constituted by fenestrae, and their numbers and membrane localization are altered within minutes^49,50^. Fenestral formation has been proposed to be orchestrated by fenestra-forming centers, which regulate the concentration of an acto-myosin ring around the new fenestrae and possibly promote depletion of intracellular organelles from the pore area^31–33^. Notably, the generation of fenestrae in liver has been proposed to be PLVAP-dependent^24^. Using a different PLVAP-deficient mouse line, Herrnberger *et al*. found a more than 6-fold reduction in the numbers of fenestrations and the absence of sieve plates in livers of 3 wk old *Plvap*^−/−^ mice. In those knock-outs, adult livers had a scarred surface, sinusoid lumens containing fibrin aggregates, a practically missing Disse’s space filled with collagen bundles, and hepatocytes full of lipid granules^24^. Moreover, severe hepatic ballooning, steatosis, leukocyte infiltration, focal necrosis and fibrosis were observed^24^. None of these alterations, which are signs of liver injury, is found in our *Plvap*^−/−^ mice. Therefore, we hypothesize that the diminished fenestrae observed in the Hernnberger-strain may be secondary due to LSEC capillarization, which is a prototypic reactive response to almost any type of liver injury^33,39,51^. Since the constructs used by Herrnberger and us are relatively similar (IRES:lacZ trapping cassette between exons 1 and 2 in the Herrnberger and loxP sites flanking the proximal promoter and in the first intron after the exon 1 in ours), the differences in the mouse strains (C57BL6N/FVB-N hybrid in the Herrnberger-strain and C57BL6N/Balb/c/NMRI hybrid in ours) may contribute to these discrepant results. In addition, the interaction of environmental factors (microbiota, food etc) with altered vascular permeability and leukocyte traffic^8,18^ induced by PLVAP-deletion may contribute to triggering of liver injury in some colonies. Although not directly analyzed in the third independent *Plvap*^−/−^ strain, Stan *et al*. do not refer to any liver injury or lack or fenestration in their mouse strain either^8^. Collectively these data indicate to us that the fenestral formation in liver is not dependent on PLVAP gene itself. Our findings in liver are in line with the observations that fenestral formation in other organs is PLVAP-independent^8^.

In addition to angiogenesis, VEGF signaling is involved in the formation of fenestrae. Trapping of hepatic VEGF diminishes fenestral formation in liver, and administration of exogenous VEGF enhances fenestral formation at least in non-hepatic vasculature^52,53^. We found that neuropilin-1, an accessory molecule enhancing VEGF signaling^48^, and the major VEGF receptor VEGFR2 are associated with PLVAP in fetal liver with diaphragmed fenestrae, but not in the adult LSEC with open fenestrae. Nevertheless, association of VEGFR2-neuropilin-1 complex with PLVAP was not necessary for the hepatic angiogenesis or fenestration, since both processes were normal in PLVAP-deficient mice. However, our findings clearly indicate that in addition to well-established homodimer formation in diaphragms^6^, PLVAP protein is capable of associating with several other surface molecules.

We found here that fenestrae in LSEC are distended by a diaphragm during the fetal development, and that the diaphragms then quite abruptly disappear at birth in mice. In rats electron microscopy studies well before the discovery of PLVAP have also reported the presence of diaphragms in fetal sinusoids in rat^36,37^, and those have also being reported in the wild-type littermate controls in the Herrnberger mouse strain^24^. The functional roles of diaphragmed fenestrae during the ontogeny are not fully understood. We have seen that the PLVAP-diaphragms regulate the exit of fetal-liver derived monocytes to blood, and thereby the seeding of fetal-derived macrophages in tissues^18^. Here we show that in fetal liver PLVAP associates with LYVE-1, VEGFR2 and neuropilin-1, suggesting that the fenestral diaphragms might regulate the localization of different supramolecular complexes during the development. In addition, the diaphragms in fetal liver sinusoids presumably restrict the movement of blood-borne molecules to the space of Disse by serving as a physicochemical filter. The signals triggering redistribution of PLVAP in LSEC at birth remain to be determined. However, they need to be quite selective since PLVAP-diaphragms distending caveolae, fenestrations and transendothelial channels were still observed in postnatal vein EC.

PLVAP has been used for decades as the golden standard marker for blood vessels^4,6,54^. The protein has thought to be present only in diaphragms, which can cover fenestrae, transendothelial channels and caveolae^6,7,55^. Therefore, PLVAP expression has been interpreted to serve as a surrogate marker for these specific nanoscale structures, which can only be detected by electron microscopy. This assumption correlates well with the fact that high PLVAP expression is seen in capillaries known to be rich in these structures (e.g. intestine, endocrine organs, choroid plexus), whereas non-leaky capillaries in the heart and brain parenchyma, and non-diaphragmed fenestrated capillaries in the glomeruli are devoid of PLVAP^6,7^. However, there has been considerable discrepancy in the literature regarding the expression of PLVAP in adult liver^8,19–24^. Our results, utilizing a validated monoclonal anti-PLVAP antibody and *Plvap*^−/−^ mice as a specificity control, now clearly show that in adult liver PLVAP is strongly expressed in the venular EC (portal and central vein), clearly expressed in sinusoidal EC and absent from the hepatic artery EC. The sinusoidal PLVAP expression reveals a novel subcellular localization of PLVAP outside the diaphragms. Moreover, our findings of similar PLVAP expression in LSEC in wild-type and Cav-1-deficient mice contradict the assumption that PLVAP would undergo rapid internalization and degradation in the absence of diaphragm-forming structures^55^.

There are limitations in our study. *Plvap*^−/−^ mice have major alterations in vascular permeability and its consequences (e.g. metabolic alterations, growth retardation) may result in secondary transcriptomic and metabolomic changes that are not directly controllable when using littermate control mice. For instance, the determinants affecting non-specific binding of MECA-32 antibody or *in vivo* intravascular availability of OVA-IC may be different between *Plvap*^−/−^ mice and their controls. Moreover, the spatial resolution of the imaging techniques used here is unable to provide definite proof for the luminal localization of PLVAP on the EC membrane outside the diaphragms, for the detailed vascular architecture or for the possible complex formation between PLVAP and LYVE-1, neuropilin and VEGFR2. Since *in situ* perfusion-fixation of embryonic liver is impossible due to ethical limitations, detailed analyses of fenestral diaphragms in embryonic LSEC by scanning or immunoelectron microscopy was not possible. However, it would be interesting to dissect the prevalence, grouping and dynamics of diaphragmed/non-diaphragmed fenestrae during physiological sieve plate formation in embryos in a future project. Nevertheless, in aggregate our current multimodal imaging data suggest that there are no apparent differences in the formation of liver vasculature or fenestrations in *Plvap*^−/−^ mice either during embryogenesis or after birth. Finally, the ambiguity regarding the expression of the prototypic leukocyte marker CD45 in LSEC.^33,56^, makes it possible that a subpopulation of LSEC may have been excluded when gating for CD45^−^ cells.

In conclusion, we have shown here using multimodal approaches that fetal liver LSEC contain fenestrae covered by PLVAP-diaphragms and that PLVAP can be engaged in novel associations with LYVE-1, VEGFR2 and neuropilin-1 in the developing liver. In contrast, adult LSEC lack diaphragms in fenestrae, but still contain cell-surface expressed PLVAP. These findings critically alter the prevailing concepts that PLVAP would only be present in diaphragms and that it would be needed for fenestral biogenesis in the liver.

## Methods

### Experimental animals

Universally and constitutively PLVAP-deficient (*Plvap*^*tm1Salm*^; hereafter designated *Plvap*^−/−^) mice and their littermate wild-type controls have been described^14^. *Cav1*^−/−^ (004585) and C57BL/6 J (000664) mice were from The Jackson Laboratory. Wild-type C57BL/6 N and BALB/c mice were from Charles River and Janvier Labs. In timed matings the day of vaginal plug appearance was considered as embryonic day 0.5 (E0.5). Postnatal analyses of *Plvap*^−/−^ mice was limited to 5 wk, since only few mice survive till early adulthood^8,9,14^.

All animal experiments were done in adherence with the rules and regulations of the Finnish Act on Animal Experimentation (62/2006) and were performed according to the 3R-principle. The experiments were approved by the Ethical Committee for Animal Experimentation in Finland (license numbers 5587/04.10.07/2014 and 6211/04.10.07/2017).

### Immunofluorescence stainings and image analysis

Livers were excised from euthanized mice and immediately cut to small pieces. The pieces were embedded in optimal cutting temperature compound (OCT; Tissue-Tek, #4583) and snap frozen using liquid nitrogen. Cryostat sections (6 μm in thickness) were cut with cryomicrotome (Leica) and fixed in ice-cold acetone.

For immunofluorescence stainings the following antibodies were used: rat anti-PLVAP primary antibody MECA-32 (BD, #550563 or BioXCell, #BE0200) detected with Alexa Fluor 488- or Alexa Fluor 647-conjugated anti-rat IgG secondary antibody (Life Technologies, #A-11006 and #A-21247, respectively), rabbit anti-LYVE-1 primary antibody (ReliaTech, #103-PA50) detected with Alexa Fluor 546-conjugated anti-rabbit IgG secondary antibody (Life Technologies, #A-11035), goat anti-neuropilin-1 primary antibody (R&D, #AF566) detected with Alexa Fluor 647–conjugated anti-goat IgG secondary antibody (Life Technologies, #A-21447), goat anti-VEGFR2 primary antibody (R&D, #AF644-SP) detected with Alexa Fluor 647–conjugated anti-goat IgG secondary antibody, APC-conjugated anti-CD31 antibody (Biolegend, #102510), Alexa Fluor 647-conjugated anti-CD144 antibody (BD, #562242) and rat anti-F4/80 primary antibody (BioRad, #MCA497GA) detected with Alexa Fluor 488- or 546-conjugated anti-rat IgG secondary antibodies (Life Technologies, #A11006 and #A11081, respectively). As a negative control for MECA-32, rat isotype control IgG2a (BD, #553926) was used. All primary and directly conjugated antibodies were diluted at predetermined optimal concentrations (5–10 μg/ml) in phosphate buffered saline (PBS; Gibco, #18912-014 or Sigma, #D8537). Secondary antibodies were diluted in PBS supplemented with 5% normal mouse serum (Jackson ImmunoResearch, #015-000-120). The sections were sequentially incubated with the indicated antibodies for 30 minutes at room temperature in a humidified chamber after which they were washed two times with PBS. Finally, the sections were mounted in ProLong Gold Antifade Mountant with or without DAPI (4,6-diaminodino-2-phenylindole; ThermoFisher Scientific, #P36931 or #P36930) or the nuclei were stained with Hoechst (diluted 1:5000 in PBS; ThermoFisher Scientific, #62249) for 5 minutes and then mounted with ProLong Gold without DAPI.

For the whole-mount stainings, the fetal livers were stained and optically cleared as described^35^.

Images were acquired using LSM 780 or LSM 880 confocal microscope with Airyscan (Carl Zeiss) equipped with c-Apochromat 40x/1.20 water objective or c-Apochromat 63x/1.20 and ZEN software or 3i Spinning disk confocal microscope equipped with a LD c-apochromat 40x/1.1 water objective and SlideBook 6 software (Intelligent Imaging Innovations).

For image analyses, background subtractions, linear brightness adjustments, and, if used, noise reductions using mean filter in ImageJ, were always applied equally to all images that were compared. Three–dimensional reconstructions were generated from *z*-stacks (wild-type: 60 slices, 0.8 μm/slice and *Plvap*^−/−^: 112 slices, 0.43 μm/slice) and the results were converted to QuickTime files with Imaris 8.0 software (Bitplane).

For the quantifications, mean gray values for MECA32 signal were measured from original 8-bit or 16-bit images using thresholded selection in the chosen areas (see Suppl. Figure 2d) using imageJ. A thresholded selection from the original image was created by first subtracting heavily Gaussian blurred background image and then applying the default thresholding method. MECA32 and CD31 signals were used for thresholding the portal vein, the three sinusoidal segments and central vein (Fig. 2d) and hepatic artery and portal vein (excluding the luminal areas; Suppl. Figure 2c and d). Resulting data was normalized to the value obtained from portal vein. Area of signal was measured from thresholded images in imageJ. Background subtraction with rolling ball method or subtracting heavily Gaussian blurred image and median filtering for noise reduction were performed prior to thresholding with Li (Fig. 3b), Otsu or Moments (LYVE-1 and MECA32 signals, respectively, Fig. 5f) methods. All steps were performed in the same way for all images that were compared with each other. Vessel diameters were determined from randomly picked LYVE-1^+^ vessels (30 vessels/genotype, 2 mice/genotype) using Image J analyses of X-Y plains of whole-mount stacks.

### Histological stainings

For hematoxylin and eosin stainings acetone-fixed cryostat sections of livers from wild-type mice were first rehydrated in H_2_O, then stained in Mayer’s Hematoxylin (Sigma, #MHS16) for 15 minutes and washed in warm tap water for 10 min. Next, the sections were dehydrated in 96% ethanol for 30 s and then stained in Eosin Y solution (Sigma Aldrich, #HT110116) for 3 min after which the excess eosin was removed by dipping the sections in absolute ethanol. Lastly, the sections were cleared in xylene (2 × 5 minutes; VWR Chemicals, #28973.363) and mounted with DPX Mountant (Sigma, #06522). The sections were imaged with Pannoramic MIDI digital slide scanner (3D Histech).

### Transmission and scanning electron microscopy and electron tomography

Transmission electron microscopy was performed as previously described^18^, except that euthanized adult mice were perfusion-fixed with 2% glutaraldehyde and 4% paraformaldehyde before the immersion fixation.

Electron tomography was performed on 150 nm sections (prepared as described above) using 15 nm gold nanoparticles deposited on both surfaces as fiducial markers. JEM-1400 Plus operated at 120 kV was used to collect dual-axis tilt series from −65° to + 65° with 1.5° steps using SerialEM version 3.3.1^57^ Tomograms were generated and visualized using the IMOD software package version 4.7.3^58^. Tomograms were filtered by the nonlinear anisotropic diffusion filter of the IMOD package.

For scanning electron microscopy tissue pieces were immersion-fixed in a phosphate buffer containing 2% glutaraldehyde and 4% paraformaldehyde (adult, but not embryonic, mice were first perfusion-fixed with the same buffer). The tissue pieces were then post-fixed for 2 h using 2% OsO4 containing 3% potassium ferrocyanide, dehydrated with a series of increasing ethanol concentrations (30%, 50%, 70%, 80%, 90%, 96% and twice 100%). Tissue pieces were then frozen by immersion in liquid nitrogen and manually fractured under liquid nitrogen^59^. The fractured pieces were immersed in hexamethyldisilazane and left to dry by solvent evaporation. Tissue pieces were coated by evaporated gold and imaged with Leo 1530 Gemini scanning electron microscope. The sieve plates and fenestral openings were manually defined from the SEM-images, and the surface areas of vessels and individual pore openings were determined using Image J.

### Immunoelectron microscopy

Mice were sacrificed with CO_2_ and transcardially perfused with 37 °C PBS, followed with perfusion of PLP-fixative containing 0.2% glutaraldehyde. Livers were collected and immersion-fixed in the same buffer for 1 h in room temperature. Samples were cryoprotected with 2.1 M sucrose in water at 4 °C overnight, embedded in OCT and snap-frozen with liquid nitrogen. 10 µm sections were cut, washed once with 0.1 M NaPO4, pH 7.4., and blocked in the same buffer containing 1% fish gelatin (Sigma # G-7765) and 1% bovine serum albumin for 30 min at room temperature. Thereafter, MECA-32 and isotype control antibodies (100 µg/ml) diluted in the washing buffer containing 0.5% fish gelatin and 0.5% bovine serum albumin were incubated on the sections at 4 °C overnight. After washings, 10 nm gold-conjugated anti-rat IgG (BBI Solutions, #EM.GAT10, 1:50 dilution) in the antibody dilution buffer was incubated on the sections at room temperature for 3 h. After washing with 0.1 M NaPO_4_, the sections were post-fixed with 2% glutaraldehyde in 0.1 M NaPO_4_ for 30 min at room temperature and washed again. The immunolabelled sections were post-fixed with 1% osmium tetroxide in 0.1 M NaPO_4_ buffer, pH 7.4, for 1 h at room temperature, washed twice, dehydrated through series of increased concentration of ethanol and acetone and gradually infiltrated into Epon resin (TAAB, Aldermaston, United Kingdom). After resin polymerization at 60 °C, 60-nm sections were cut (EM Ultracut UC6i ultramicrotome, Leica Mikrosysteme GmbH, Austria) and post-stained with uranyl acetate and lead citrate.

### Detection of cell-surface PLVAP

For confocal analyses, 10 µg of MECA-32 was intravenously injected to tail vein of 4 wk old wild-type and *Plvap*^−/−^ mice. After 10 min, mice were sacrificed using CO_2_ and livers were collected, cut to small pieces, embedded in OCT and snap frozen using liquid nitrogen. Acetone-fixed frozen sections were then stained *ex vivo* with anti-rat IgG-A647 (to detect *in vivo* bound MECA-32) followed by rabbit anti-mouse LYVE-1 and A546-conjugated anti-rabbit IgG secondary antibody. In control stainings the second-stage antibodies were confirmed to be completely species-specific.

For electron microscopy analyses, MECA-32 antibody was directly conjugated to 10 nm gold nanoparticles using GOLD conjugation kit (Abcam, #201808), and the buffer was changed to PBS. Conjugated MECA-32 (5 µg) was intravenously injected to a C57Bl/6 N wild- type female mouse. After 10 min circulation time, the mouse was sacrificed with CO_2_ and perfusion-fixed by transcardial perfusion of 0.2 M phosphate buffer containing 4% paraformaldehyde and 2% glutaraldehyde. Thereafter, the liver was collected and processed as described above for transmission electron microscopy.

### Immunoblottings

Immunoblotting was performed as previously described^18^. In brief, at autopsy, whole livers were dissected from wild-type, *Plvap*^−/−^ and *Cav1*^−/−^ mice, chopped into small tissue fragments mechanically using scissors, and lysed for 1 h at 4 °C in a buffer containing 0.2% NP-40, 150 mM NaCl, 1.5 mM MgCl_2_, 10 mM Tris, pH 7.2, and Roche Protease Inhibitor Cocktail. After clarification by centrifugation, the protein concentrations were measured using Bio-Rad DC kit from the lysate supernatants. Aliquots of lysates were mixed with non-reduced Laemmli’s sample buffer and heated to 37 °C for 10 min. The samples were then resolved in SDS-PAGE (5 µg protein/lane; under non-reducing conditions). After transfer to nitrocellulose, PLVAP was visualized with MECA-32 using an enhanced chemiluminescence detection system (Amersham). After stripping the same filter was reprobed for GAPDH expression. The band quantifications were made using ImageJ. Equal ROIs were selected and mean grey values were calculated. Thereafter background value (an irrelevant area in image) was subtracted from these, and finally the PLVAP/GAPDH signal ratio was determined. The original full-size immunoblots are shown in Suppl. Figure 10.

### *In vivo* scavenging assays

Particles, modified proteins and immunocomplexes were administered to adult *Plvap*^−/−^ and control mice. As particles, 20 µl of 0.5 µm FluoSpheres carboxylate-modified microspheres (505/515; ThermoFisher, #F8813) and 0.02 µm FluoSpheres carboxylate-modified microspheres (660/680; ThermoFisher, #F8783) were used. Alexa Fluor 594-conjugated acetylated-LDL (ThermoFisher, #L35353; 10 µl/mouse) represented modified proteins. To prepare ovalbumin (OVA)-anti-OVA antibody immunocomplexes, Atto488-conjugated OVA (Sigma, #41235) or Alexa Fluor 647-conjugated OVA (Q34784, Molecular probes) (50 µl from 2 mg/ml solution in PBS) was mixed with an rabbit polyclonal anti-OVA IgG (5.4 µl from 3.7 mg/ml solution, Sigma, #C6534) for 1 h at 4 ^o^C, and 55.4 µl of the complex was then administered per mouse. All compounds were injected into tail vein and allowed to circulate for 2 h before collecting the livers for microscopy and FACS analyses. Only mice in which the total injection volume was smoothly delivered were included in the analyses.

### Flow cytometry and sortings

Parenchymal liver cells in untouched or OVA-immunocomplex treated wild-type and *Plvap*^−/−^ mice (see above) were dissociated using Gentle MACS C-tube (Miltenyi Biotech) and enriched using 26.6% Optiprep gradient. The cells were first stained with Fixable viability dye eFluor780 (eBioscience, #65–0866) according to the manufacturer’s instructions to discriminate dead and live cells. Next, the cells were incubated with purified anti-CD16/32 for 10 min on ice to block non-specific binding to Fc-receptors. The cells were then stained for 30 min on ice using fluorochrome-conjugated monoclonal antibodies. The endothelial cell panel included PerCP-Cy5.5-conjugated or V500-conjugated CD45 (BD, #550994 and 561487, respectively), Alexa-647-conjugated CD144 (BD, # 562242), PE-conjugated Lyve-1 (R&D Systems, FAB2125P); Alexa-488 conjugated MECA-32 (Biolegend, #120506) and PE-Cy7-conjugated podoplanin (Biolegend, # 127412). The macrophage panel included CD45, V500-conjugated CD45 (BD, #561487), BV786-conjugated CD11b (BD, #740861) and Alexa Fluor 647-conjugated F4/80 (BioRad, #MCA497A647) antibodies. All FACS analyses were run using LSRFortessa flow cytometer (BD Biosciences) and analyzed using FlowJo (Tree Star Inc.) software.

For sortings, viable (viability dye negative) liver sinusoidal (CD45^−^Podoplanin^−^LYVE-1^+^CD144^+^) and non-sinusoidal (CD45^−^ Podoplanin^−^LYVE-1^−^CD144^+^) vascular EC were isolated using FACS aria II (70 µm nozzle, Beckton-Dickinson) cell sorters. The purity of the isolated populations was >95%.

### Quantitative real-time PCR

Total RNA was isolated from sorted liver EC (see above) using RNeasy Plus Micro Kit (QIAGEN, #74034) and reverse transcribed to cDNA with SuperScript VILO cDNA Synthesis Kit (ThermoFisher Scientific, #11754050) according to the manufacturers’ instructions. Quantitative PCR was carried out using Taqman Gene Expression Assays (ThermoFisher Scientific) for *Plvap* (Mm00453379_m1; target gene), *Lyve-1* (Mm00475056_m1; target gene) and *Actb* (Mm00607939_s1; control gene).

### Blood and serum analyses

Cardiac blood was drawn from 5 wk old wild-type and *Plvap*^−/−^ mice into heparinized tubes. Automated hemocytometer (VetSCan HM 5, Abaxis) was used to determine the blood erythrocyte, platelet, total leukocyte, lymphocyte, neutrophil and monocyte numbers. The total protein, albumin, amylase and blood urea nitrogen concentrations were analyzed from the heparinized plasma using VetScan VS2 (Abaxis).

### Proximity ligation assays (PLA)

To study PLVAP-nrp1, PLVAP-LYVE1, PLVAP-VEGFR2 and VEGFR2-nrp1 associations, proximity ligation assays (PLA) were performed^46^. Briefly, primary antibodies to VEGFR2 (R&D, #AF644-SP) and LYVE1 were detected using Duolink *in situ* PLA probe anti-goat PLUS (Sigma, #DUO92003) and PLA probe anti-rabbit PLUS (Sigma, #DUO92002), respectively. Rat anti-PLVAP antibody (MECA-32) and goat anti-neuropilin1 were directly conjugated to Duolink *in situ* PLA probe MINUS using Duolink *in situ* probemaker MINUS kit (Sigma, #DUO92010). After antibody stainings and PLA probe incubations, the ligation and amplifications were performed using Duolink *in situ* Detection kit red (Sigma, #DUO92008). During the amplification step, Alexa 488-conjugated donkey anti-rat IgG (Invitrogen, #A21208) and Alexa647-conjugated donkey anti-goat IgG (Invitrogen, #A21447) were added to detect PLVAP and neuropilin1/VEGFR2, respectively. Nuclei were stained with DAPI and the slides were mounted in Mowiol. Images were obtained using a 3i Spinning disk confocal microscope equipped with a plan-apochromat 63x/1.4 oil objective and SlideBook 6 software (Intelligent Imaging Innovations) or LSM780 (Carl Zeiss) equipped with c-Apochromat 40x/1.20 water objective and using ZEN software.

ImageJ was used for quantifications. Background was subtracted using rolling ball method for the PLA-signal and sinusoidal signal (sinusoids identified by MECA-32-positivity or with nrp1/ VEGFR2-positivity, as appropriate). Sinusoidal ROI area was generated by thresholding the sinusoidal signal. After applying ROIs to thresholded PLA-signal, % of area was measured and quantified from 3–5 images/mouse (63x objective) and 2 images/mouse (40x objective), respectively. All adjustments were applied equally for the samples that were compared.

### Statistical analysis

Comparisons between genotypes or treatments were done with Mann-Whitney U-test using Graphpad Prism 7 or SAS version 9.4 software. All quantitative data are given as mean ± sem. P values of <0.05 were considered to be statistically significant.

## Supplementary information

Supplementary material

Supplemental video

## Data Availability

All materials are available commercially or from the authors. All data generated or analysed during this study are included in this published article (and its Supplementary Information files).
