# Supplementary material for: Fenestral diaphragms and PLVAP associations in liver sinusoidal endothelial cells are developmentally regulated
Source: Sci Rep. 2019 Oct 30;9:15698. doi: 10.1038/s41598-019-52068-x (PMC6821839; doi:10.1038/s41598-019-52068-x)
Supplement: Supplementary file 1 — Supplementary material [file 41598_2019_52068_MOESM1_ESM.pdf]

Supplemental materials for

Fenestral diaphragms and PLVAP associations in liver sinusoidal endothelial cells are developmentally regulated

Kaisa Auvinen, Emmi Lokka, Elias Mokkala, Norma Jäppinen, Sofia Tyystjärvi, Heikki Saine, Markus Peurla, Shishir Shetty, Kati Elima, Pia Rantakari, Marko Salmi

Contents:

Supplemental Video Legend 1

Supplemental Figure Legends

Supplemental Figures 1-10

Supplemental Video legend

Supplemental video 1. Whole-mount imaging of liver vasculature for LYVE1 and PLVAP. Optically cleared E14.5 livers from wild-type (left) and *Plvap*$^{-/-}$ (right) mice were stained for LYVE1 and PLVAP. Shown are three-dimensional reconstructions from a 48 µm thick volume of the livers. The first 12 s of the video shows the LYVE-1 signal only (gray), and then the PLVAP signal (green) is superimposed for the latter half of the video (note that the *Plvap*$^{-/-}$ specimen was also identically stained for the PLVAP but as expected it lacks the reactivity). Videos are representative from n=2 mice/genotype.

Supplemental Figure legends

Supplemental Figure 1. PLVAP and diaphragms in the vasculature of adult liver. **a** Confocal microscopy analyses of PLVAP and LYVE-1 expression in LSEC in 5 wk old wild-type mice of the indicated strains. Bars, 10 µm. **b** Confocal microscopy analyses of LSEC in 5 wk old wild-type mice of the indicated strains stained for LYVE-1 and an isotype-matched control mAb for PLVAP. Bars, 10 µm. **c** Low-magnification transmission electron micrographs from a vein in liver of a 9 wk old wild-type mouse. VL, vascular lumen, BM, basement membrane. Red arrows, diaphragms in caveolae, fenestrae, and transendothelial channels. Bars, 10 µm (left), 200 nm (right). The boxed areas are shown at a higher magnification in the main Fig. 2a. **d,e** Transmission electron micrographs from a vein in liver of a 5 wk old *Plvap*$^{-/-}$ mice. VL, vascular lumen, BM, basement membrane. The two boxed areas are shown at a higher magnification in the insets. Red arrowheads, endocytic vesicles/caveolae and fenestrae or transendothelial channels without diaphragms. Bar, 200

nm (40 nm in the insets). Shown are representative images (numbers of mice/genotype: n=3(a-e).

Supplemental Figure 2. Phenotypic heterogeneity of vascular EC in liver. **a-c** Analysis of PLVAP expression in hepatic artery and portal vein in liver. **a** Hematoxylin-eosin staining of a portal tract. Arrows point to arteries. PV, portal vein, BD, bile duct. **b** Confocal immunofluorescence staining of a consecutive section (from a) for PLVAP and CD31. Arteries (yellow arrowheads) are identified by CD31-positivity. PV, portal vein, BD, bile duct. Bar, 50 µm. **c** Quantification of relative PLVAP staining intensities (mean±s.e.m; 3 mice/genotype) from the portal vein and artery. Intensity value 1.0 was assigned to the portal vein in each animal. **d**, Definition of the areas used for the quantification of PLVAP signal intensity for main Fig. 2d. 1, portal vein, 2, periportal 50 µm zone, 3, midlobular, 4, pericentral 50 µm zone, and 5, central vein. Note that the non-cellular lumen of the portal and central vein was excluded by thresholding from the analyses (see the methods for details). **e,f** Low-magnification images of liver sinusoids stained for PLVAP, LYVE-1 and CD144 (**e**) and PLVAP, LYVE-1 and CD31 (**f**). White arrowheads point to LYVE-1^{-}PLVAP^{+}CD144^{+} (e) and LYVE-1^{-}PLVAP^{+}CD31^{+} (f) sinusoids. Red arrowheads point to LYVE-1^{+}PLVAP^{+}CD144^{+} (e) and LYVE-1^{+}PLVAP^{+}CD31^{+} sinusoids (f). Bars, 50 µm. Shown are representative images from n=3 mice/genotype (a,b,e,f).

Supplemental Figure 3. Adult caveola-deficient and aged wild-type mice express sinusoidal PLVAP. **a** Confocal microscopy analyses of PLVAP-1 and LYVE-1 expression in 5 wk old wild-type and *Cav1*$^{-/-}$ mice. CV, central venule; PV, portal venule. Bars, 50 µm. **b,c** Higher magnifications of the central and periportal areas (the green boxes 1 and 3) in (a). Bars, 10 µm. The higher magnification of the mid-lobular segment is shown in the main Fig.

3c. **d** Confocal microscopy analyses of PLVAP-1 and LYVE-1 expression in 24 wk old wild-type mice. CV, central venule; PV, portal venule. Bars, 100 µm. **e** Higher magnifications of the central and periportal areas (the green boxes 1 and 3) in (d). Bars, 10 µm. The higher magnification of the mid-lobular segment (box 2) is shown in the main Fig. 3d. Shown are representative images (numbers of mice/genotype: n=4-6 (a-c), n=1 (d,e)

Supplemental Figure 4. PLVAP is expressed on the surface of adult LSEC in the absence of diaphragms. **a,b** Low-magnification immuno-electron micrographs of liver sections stained with MECA-32 (**a**) and rat IgG2a isotype control (**b**) antibody followed by an anti-rat immunoglobulin conjugated to 10 nm gold particles. Higher magnifications of the boxed areas are shown in the main Figs. 4b and c. E, sinusoidal endothelial cell; SD, space of Disse, H,hepatocyte. Bars, 40 nm. **c** Electron micrograps of LSEC in 5 wk old wild-type mouse 10 min after an intravenous bolus of the anti-PLVAP-1 antibody (MECA-32) directly conjugated to 10 nm gold nanoparticles. Higher magnifications of the boxed area are shown in the insets. E, sinusoidal endothelial cell; SD, space of Disse, H,hepatocyte. Bars, 40 nm. Shown are representative images (numbers of mice: n=3 (a,b), n=1 (c).

Supplemental Figure 5. Vascular architecture in liver is intact in the absence of PLVAP. **a** Low magnification electron migrographs from the sinusoidal vessels in 5 wk old wild-type and *Plvap*$^{-/-}$ mice. Bars, 2 µm. The width of space of Disse is marked by the red line. Quantitative data are mean±s.e.m. (6-10 sinusoids/genotype, 2 mice/genotype; the widths were determined as the average of 10-18 measurements/sinusoid). **b-e** Confocal microscopy analyses and quantification of CD31^{+} area (mean±s.e.m, 4 mice/genotype) of LYVE-1 and CD31 expression in the livers of 5 wk old wild-type and *Plvap*$^{-/-}$ mice. Higher magnifications from the boxed pericentral, mid-lobular and periportal segments of (**b**) are

shown in (**c**-**e**)). CV, central venule; PV, portal venule. Bars, 100 µm (10 µm in the insets). **f** Gating strategy for flow cytometric analysis of liver EC (for main Fig. 5c). Leukocytic ($CD45^+$) cells were excluded from live, singlet cells, and the non-leukocytic cells were analyzed for the expression of the endothelial markers LYVE1 and CD144. **g** Scanning electron microscopy analyses of immersion-fixed E17.5 livers from wild-type and *Plvap*$^{-/-}$ mice. The arrows point to representative putative fenestrae in vessels. Bars, 1 µm. Shown are representative images from 3 (b-d) or 1 (g) mice/genotype.

Supplemental Figure 6. Scavenging of different molecules by liver sinusoidal endothelial cells and macrophages in wild-type and *Plvap*$^{-/-}$ mice. Acetylated LDL and 0.02 µm particles (**a and c**) and 0.5 µm particles and ovalbumin immunocomplexes (OVA-IC) (**b and d**) were injected intravenously to wild-type and *Plvap*$^{-/-}$ mice, and their distribution in liver was analyzed 2 h later. The liver sections were stained with LYVE-1 (a and b) or F4/80 (c and d) and DAPI. Bars, 50 µm (main figure) and 10 µm (the zoom-ins). Shown are representative images (n=3-4 mice/genotype)

Supplemental Figure 7. Scavenging of ovalbumin-immunocomplexes (OVA-IC) by liver macrophages in wild-type and *Plvap*$^{-/-}$ mice. **a**, The gating strategy for identification of Kupffer and non-Kupffer macrophages in the liver. The cells were sequentially gated based on scatters, viability (live cell marker), singlets, CD45, CD11b and F4/80. **b**, The numbers of Kupffer and non-Kupffer macrophages in liver. (**c-e**) OVA-IC was injected intravenously, and after 2 h the binding/uptake in the two macrophage populations was determined by flow cytometry. **c** Representative histograms for OVA-IC expression. **d** The percentage of Kupffer cells positive for OVA-IC. **e** Mean fluorescence intensity (MFI) of OVA-IC expression. In the quantifications, each dot represents one mouse. * $p<0.05$.

Supplemental Figure 8. Characterization of *Plvap*$^{-/-}$ mice. **a** Blood cell composition of analyzed by Vetscan. **b** Representative photographs from plasma. **c** Blood protein concentrations. **d** Weights of the living animals. **e** Wet weights of the isolated livers and the liver weight/animal weight ratios. All data in a-e are mean±sem, and each dot represents one mouse. The total protein and albumin concentrations in *Plvap*$^{-/-}$ mice were below the detection limit of the assay. In all experiments 5 wk old *Plvap*$^{-/-}$ and sex-matched littermate wild-type mice were compared.

Supplemental Figure 9. Expression of neuropilin-1 and VEGFR2 in the liver vasculature. **a,b** Confocal microscopy analyses of LYVE-1 and neuropilin-1 (nrp1) in wt and *Plvap*$^{-/-}$ mice at E14.5 (**a**) and 5 wk (**b**). Bars, 10 µm. **c** Confocal microscopy analyses of PLVAP and VEGFR2 in wt mice at E14.5 and 5 wk. Bars, 10 µm. Shown are representative images from 3 mice/genotype.

Supplemental Figure 10. The original images of immunoblots. Shown are the original uncropped immunoblots for Fig. 1c PLVAP (**a**), Fig.1c GAPDH (**b**), Fig. 3a PLVAP (**c**) and Fig. 3a GAPDH (**d**). The lane labelings in b-d are not visible on these films since they were marked on films from different exposure times. The black boxes indicate the areas depicted in the main figures.

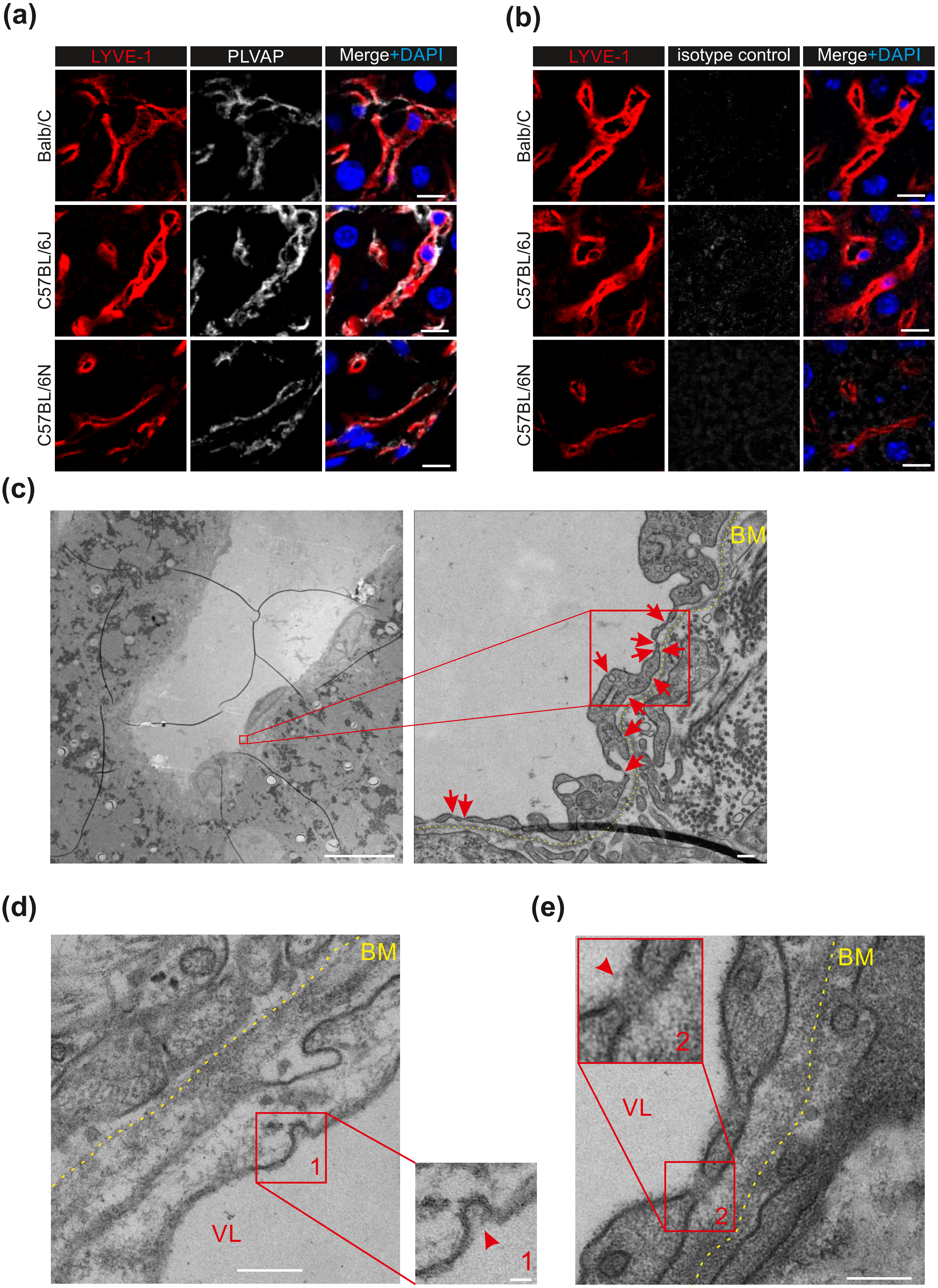

(a)
LYVE-1
PLVAP
Merge+DAPI
Balb/C
C57BL/6J
C57BL/6N
(b)
LYVE-1
isotype control
Merge+DAPI
Balb/C
C57BL/6J
C57BL/6N
(c)
BM
(d)
BM
1
VL
1
(e)
2
BM
VL
2

FIG. S1

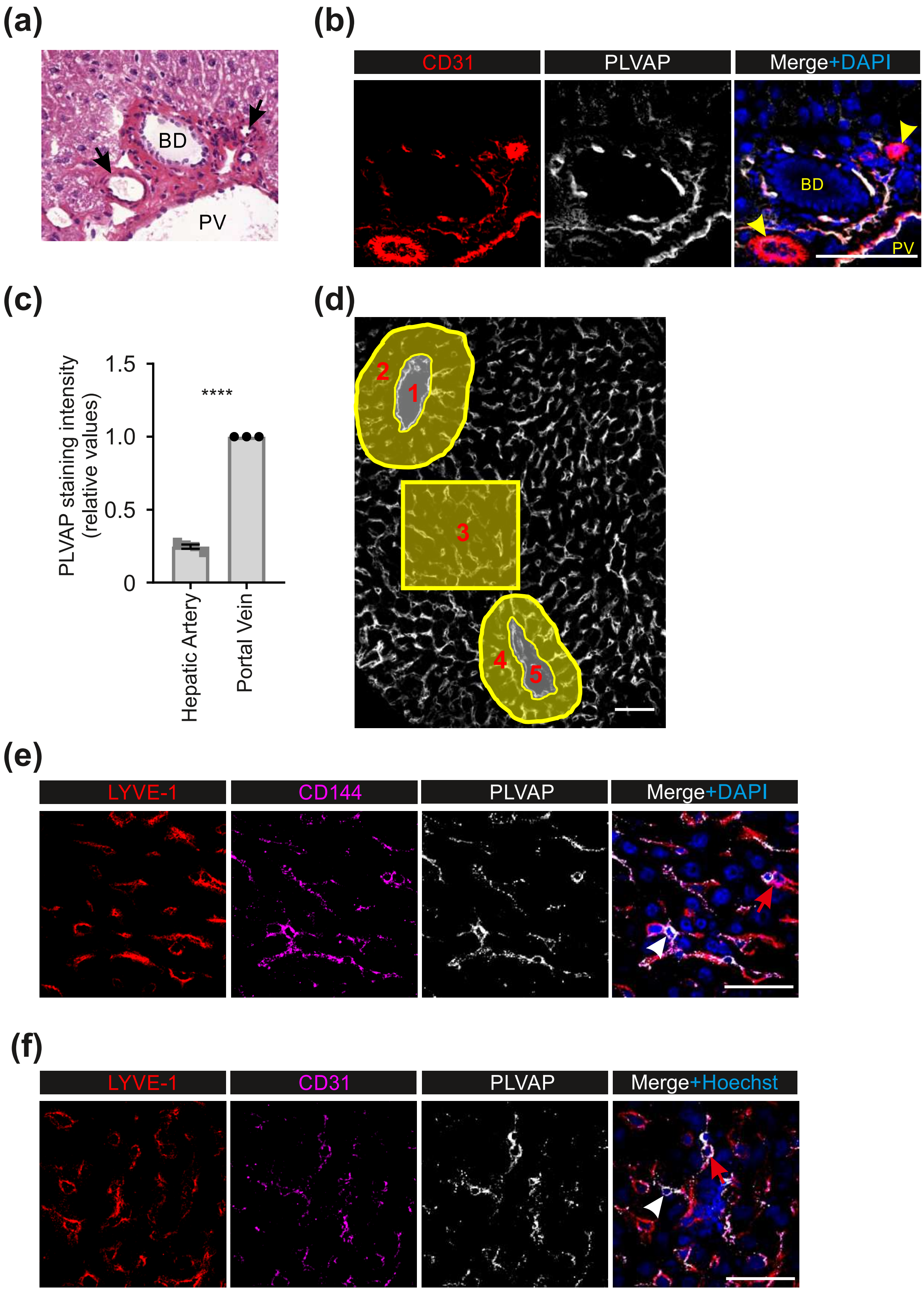

(a)
BD
PV
(b)
CD31
PLVAP
Merge+DAPI
BD
PV
(c)
PLVAP staining intensity (relative values)
1.5
1.0
0.5
0
****
Hepatic Artery
Portal Vein
(d)
1
2
3
4
5
(e)
LYVE-1
CD144
PLVAP
Merge+DAPI
(f)
LYVE-1
CD31
PLVAP
Merge+Hoechst

FIG. S2

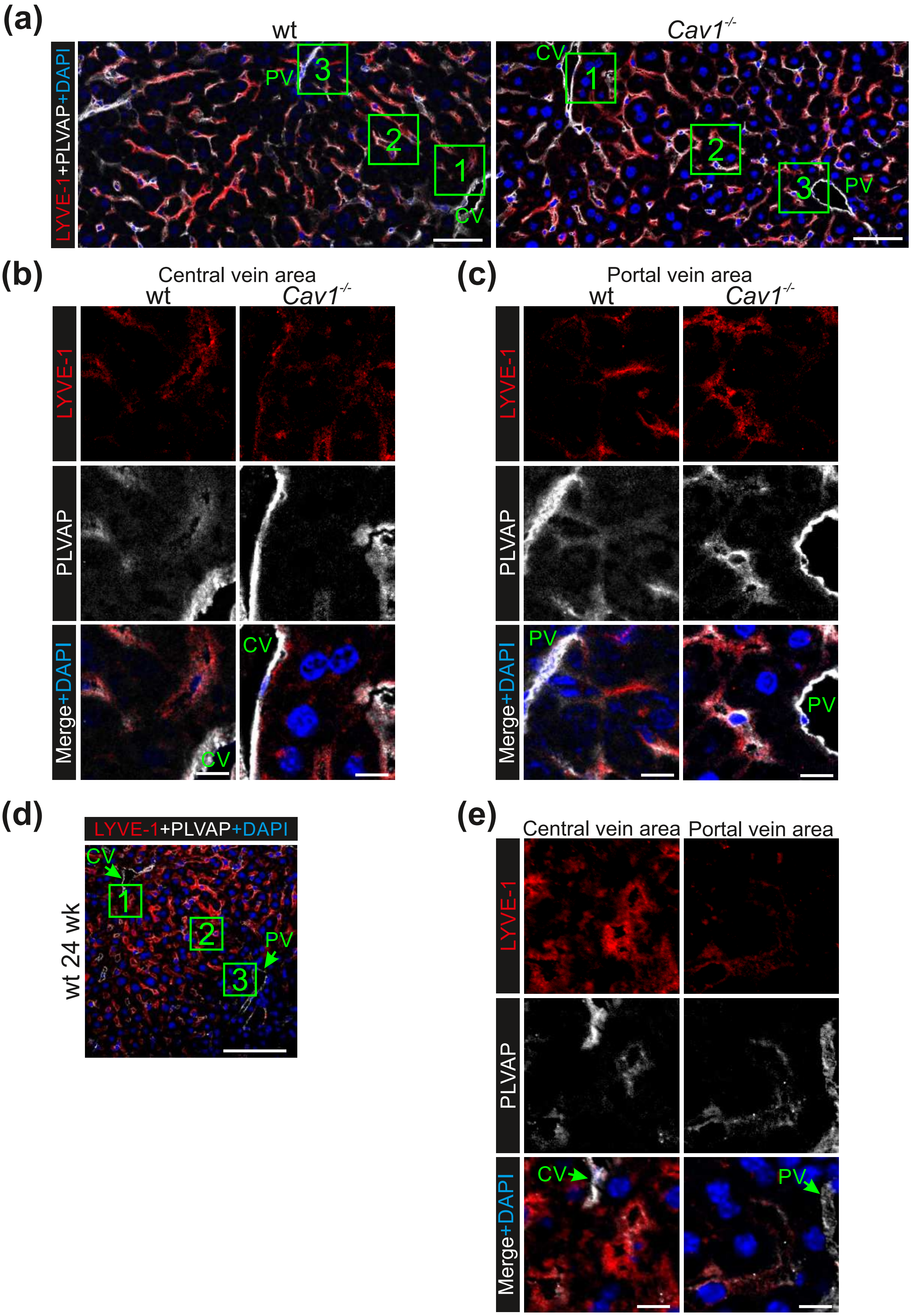

(a)
wt
Cav1-/-
LYVE-1+PLVAP+DAPI
PV
CV
1
2
3
(b)
Central vein area
wt
Cav1-/-
LYVE-1
PLVAP
Merge+DAPI
CV
(c)
Portal vein area
wt
Cav1-/-
LYVE-1
PLVAP
Merge+DAPI
PV
(d)
LYVE-1+PLVAP+DAPI
wt 24 wk
CV
PV
1
2
3
(e)
Central vein area
Portal vein area
LYVE-1
PLVAP
Merge+DAPI
CV
PV

FIG. S3

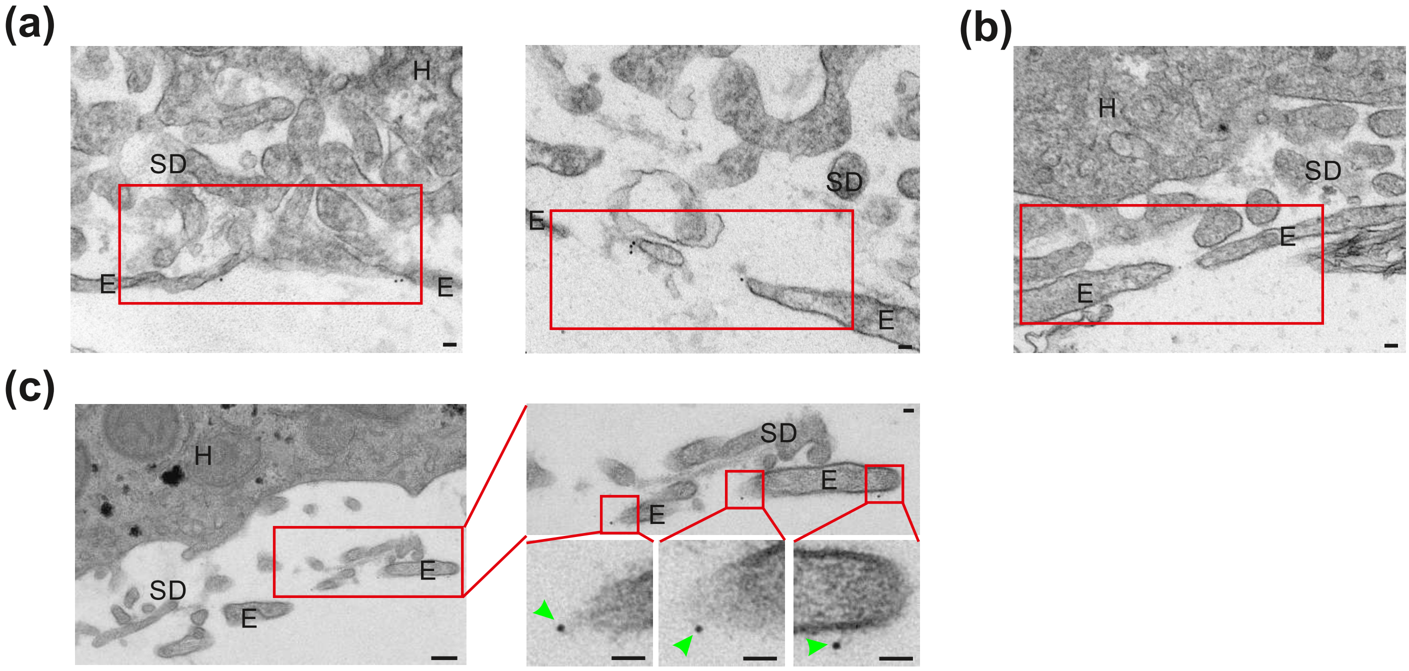

FIG. S4

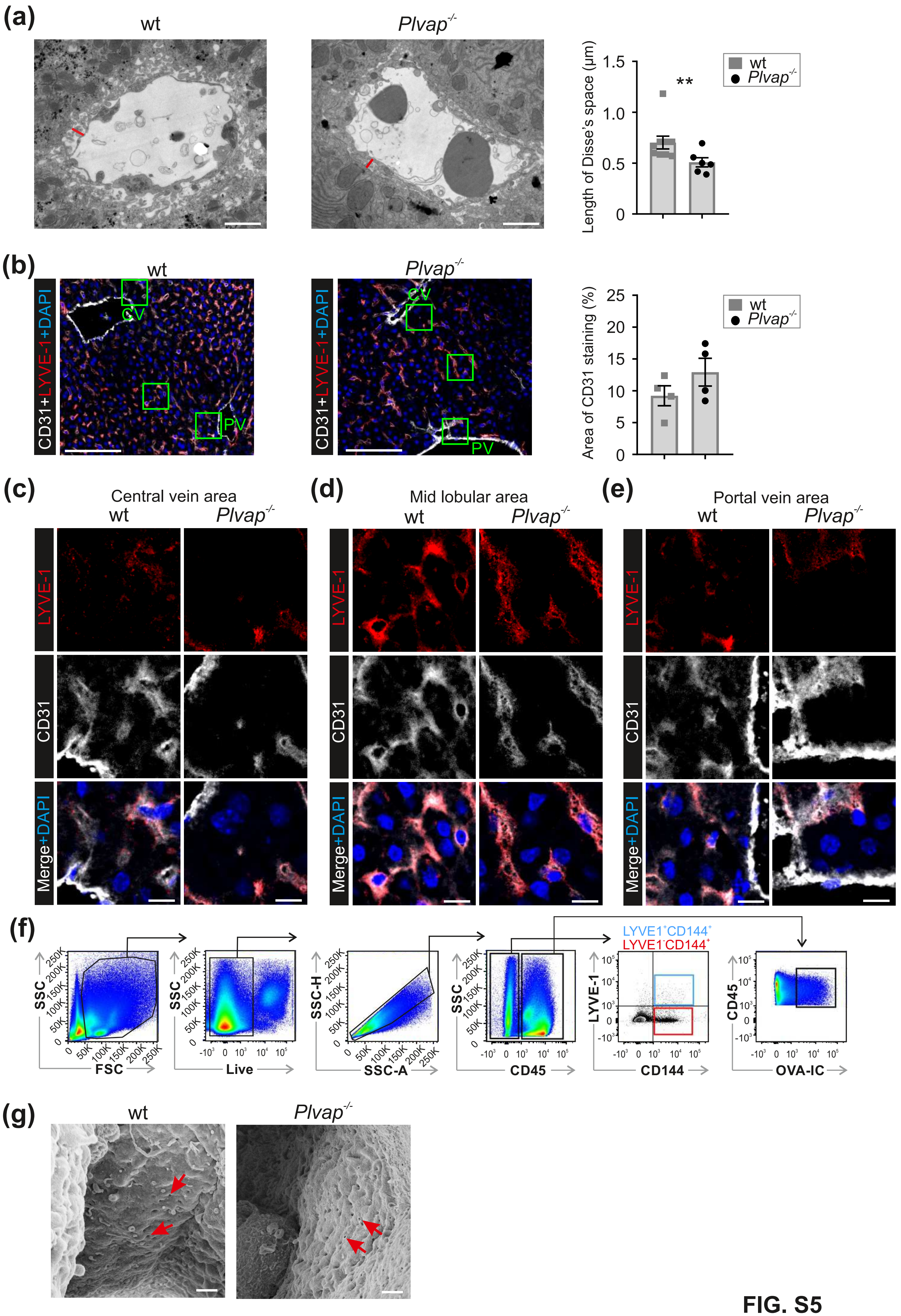
(a)
wt
Plvap-/-
Length of Disse's space (µm)
wt
Plvap-/-
**
(b)
CD31+LYVE-1+DAPI
CV
PV
Area of CD31 staining (%)
(c)
Central vein area
(d)
Mid lobular area
(e)
Portal vein area
LYVE-1
CD31
Merge+DAPI
(f)
SSC
FSC
Live
SSC-H
SSC-A
CD45
LYVE1+CD144+
LYVE1-CD144+
LYVE-1
CD144
OVA-IC
(g)

FIG. S5

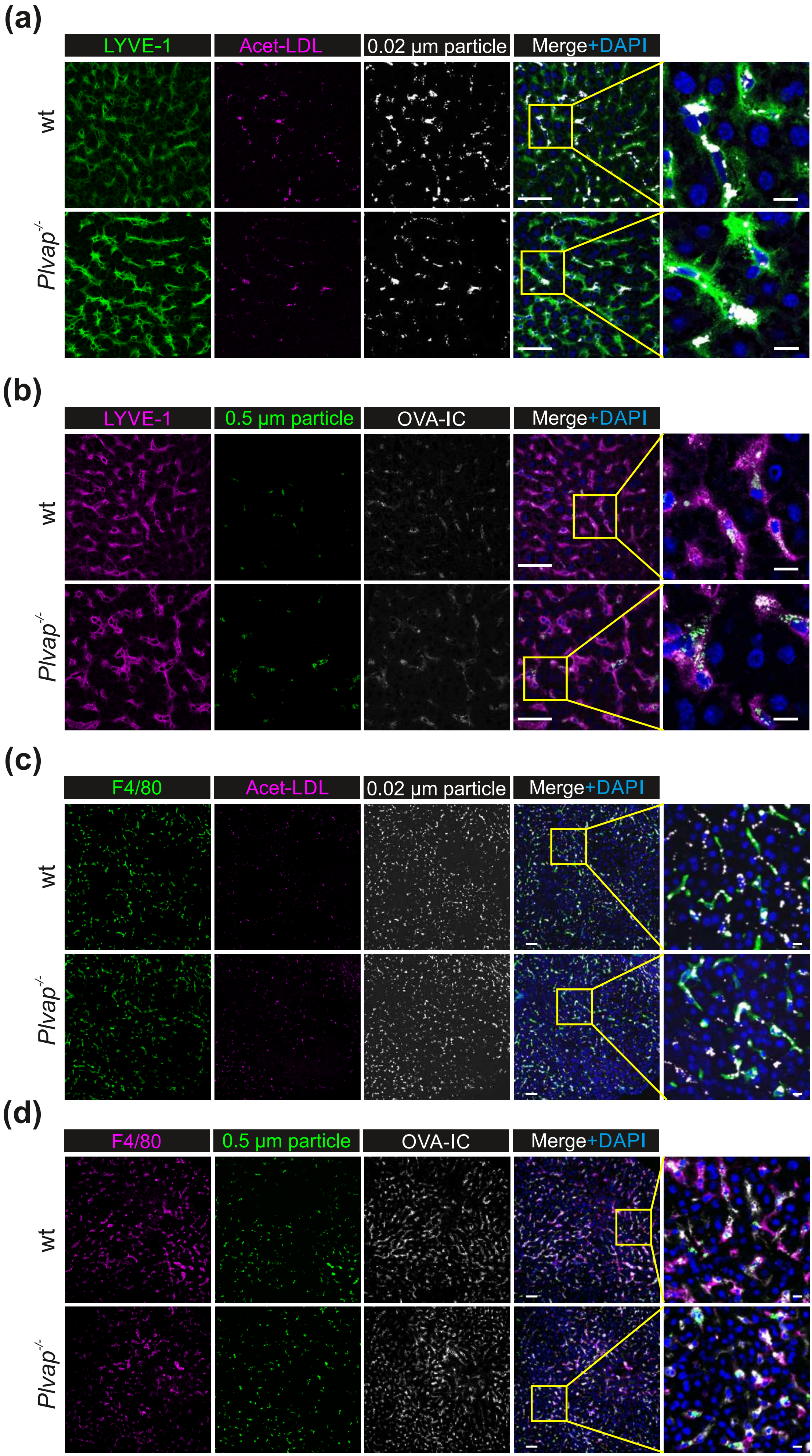

(a)
LYVE-1
Acet-LDL
0.02 µm particle
Merge+DAPI
wt
Plvap-/-
(b)
LYVE-1
0.5 µm particle
OVA-IC
Merge+DAPI
wt
Plvap-/-
(c)
F4/80
Acet-LDL
0.02 µm particle
Merge+DAPI
wt
Plvap-/-
(d)
F4/80
0.5 µm particle
OVA-IC
Merge+DAPI
wt
Plvap-/-

FIG. S6

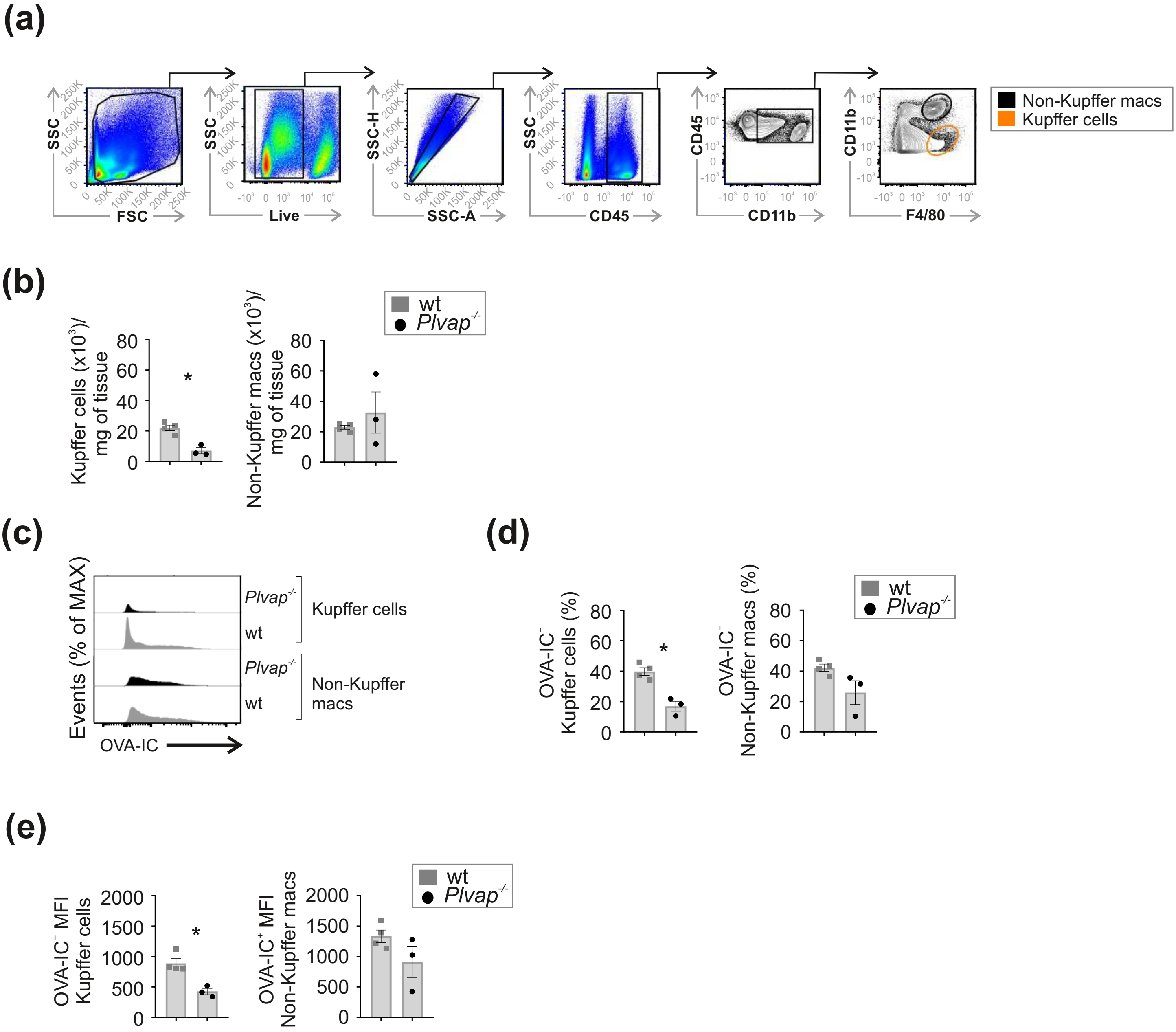
(a)
SSC
FSC
Live
SSC-H
SSC-A
CD45
CD11b
F4/80
Non-Kupffer macs
Kupffer cells
(b)
wt
Plvap-/-
Kupffer cells (x10³)/ mg of tissue
Non-Kupffer macs (x10³)/ mg of tissue
(c)
Events (% of MAX)
OVA-IC
Plvap-/-
wt
Kupffer cells
Non-Kupffer macs
(d)
OVA-IC+ Kupffer cells (%)
OVA-IC+ Non-Kupffer macs (%)
(e)
OVA-IC+ MFI Kupffer cells
OVA-IC+ MFI Non-Kupffer macs

FIG. S7

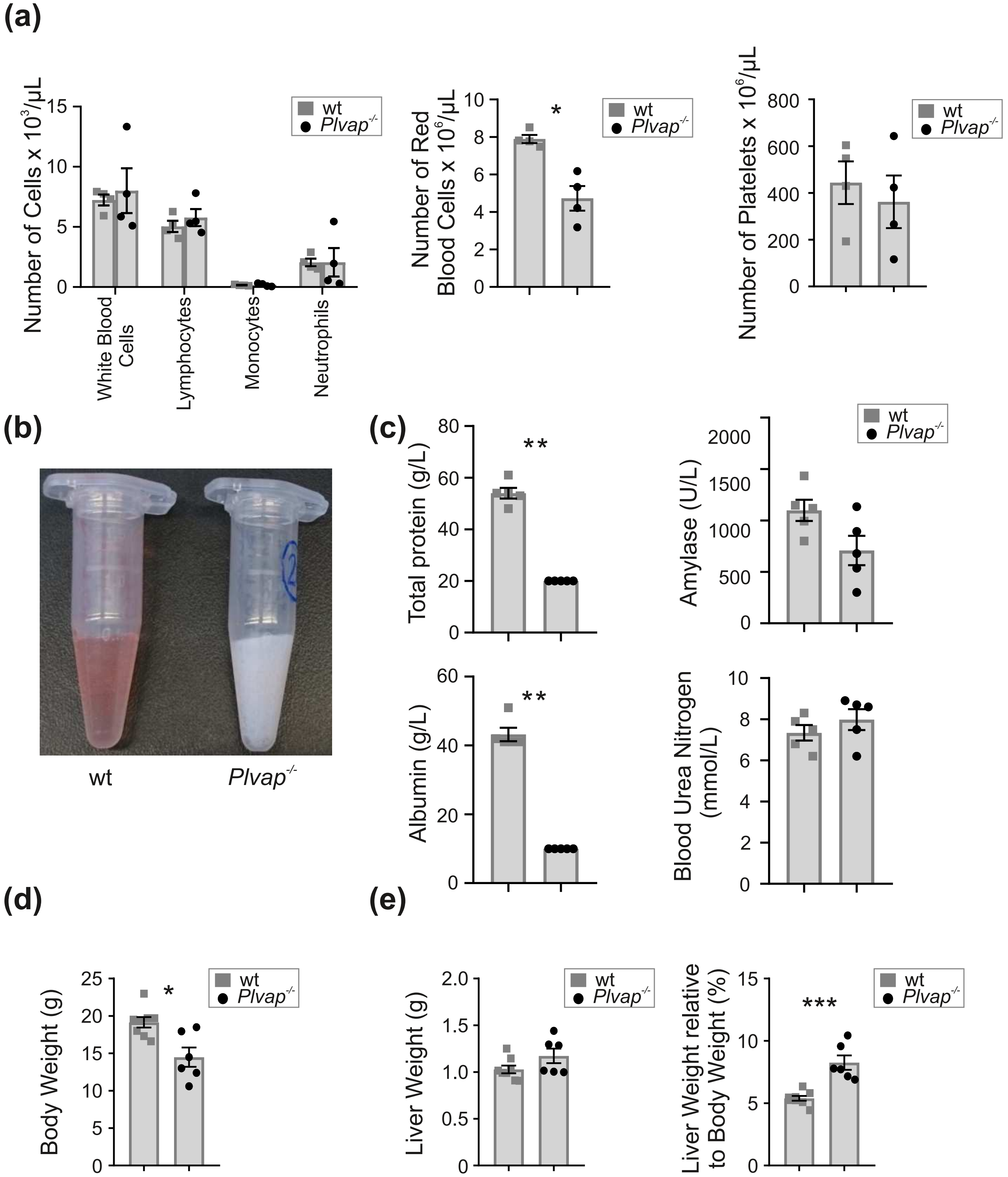
(a)
Number of Cells x 10^3/µL
White Blood Cells
Lymphocytes
Monocytes
Neutrophils
wt
Plvap-/-
Number of Red Blood Cells x 10^6/µL
*
Number of Platelets x 10^6/µL
(b)
wt
Plvap-/-
(c)
Total protein (g/L)
**
Amylase (U/L)
Albumin (g/L)
**
Blood Urea Nitrogen (mmol/L)
(d)
Body Weight (g)
*
(e)
Liver Weight (g)
Liver Weight relative to Body Weight (%)
***

FIG. S8

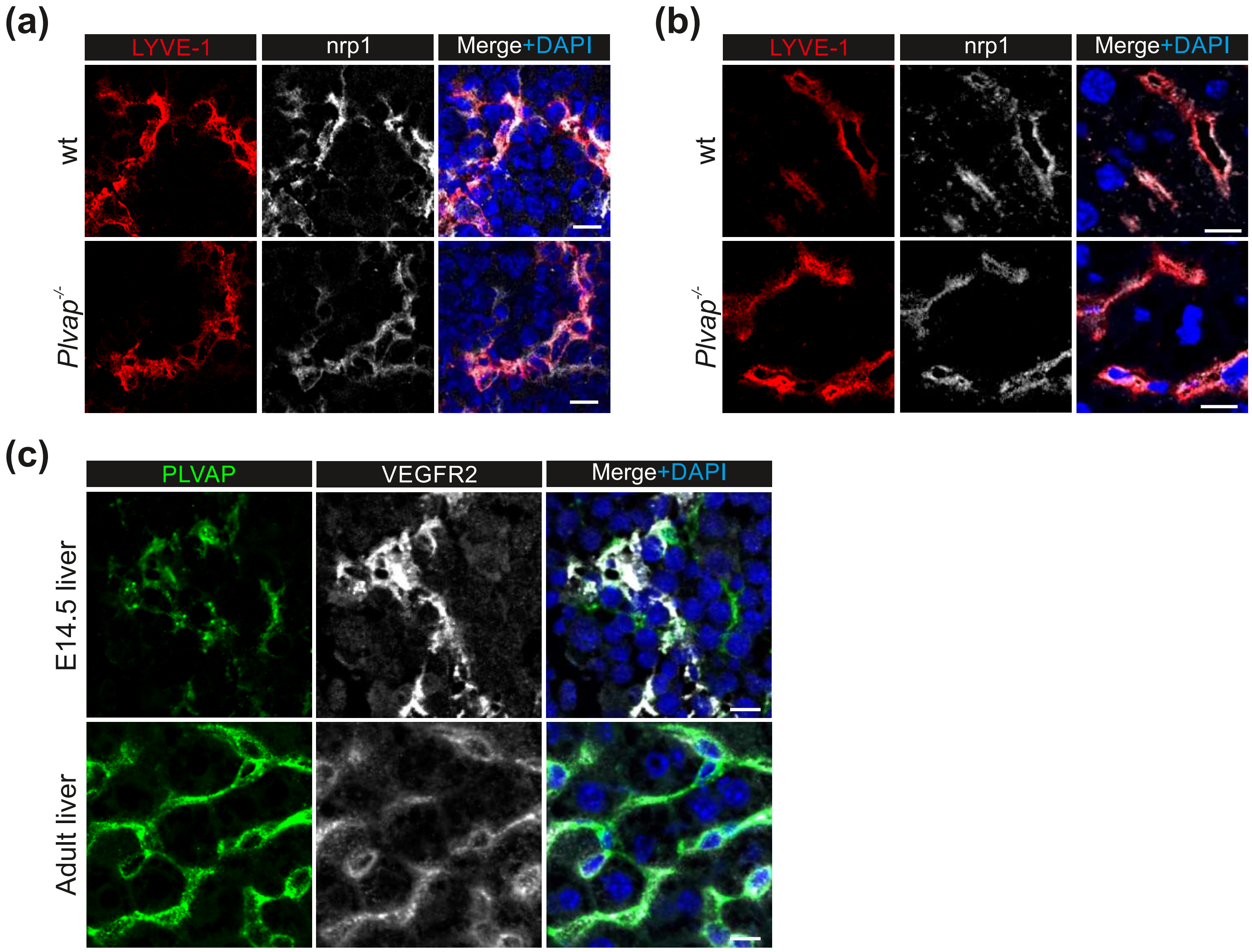
(a)
LYVE-1
nrp1
Merge+DAPI
wt
Plvap-/-
(b)
LYVE-1
nrp1
Merge+DAPI
wt
Plvap-/-
(c)
PLVAP
VEGFR2
Merge+DAPI
E14.5 liver
Adult liver

FIG. S9

(a)

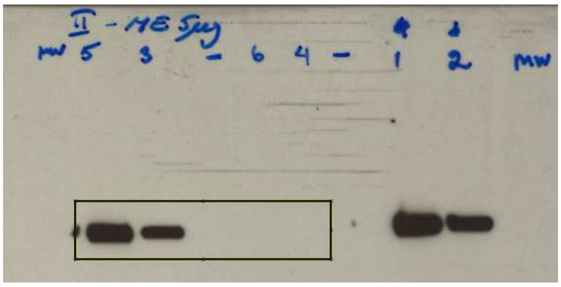

(b)

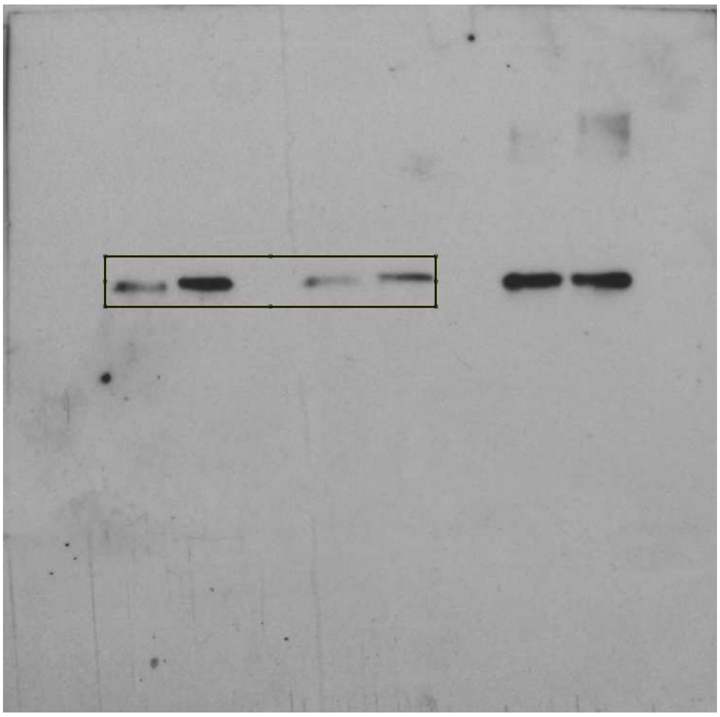

(c)

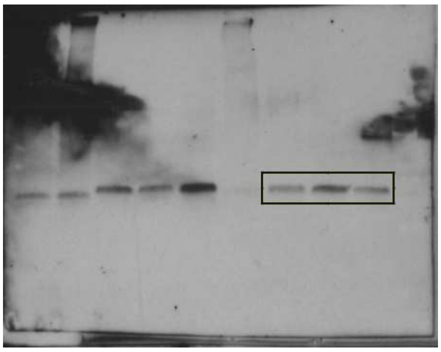

(d)

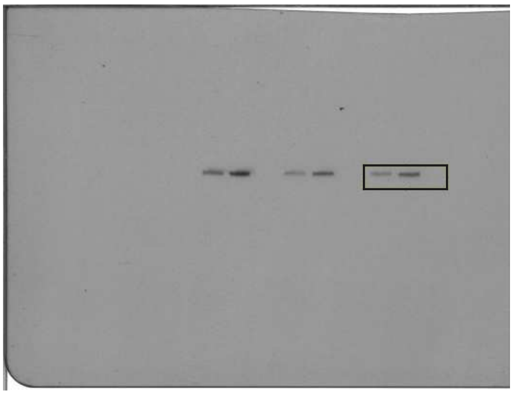

FIG. S10
